# Structural basis for recognition of the tumor suppressor protein PTPN14 by the oncoprotein E7 of human papillomavirus

**DOI:** 10.1371/journal.pbio.3000367

**Published:** 2019-07-19

**Authors:** Hye-Yeoung Yun, Min Wook Kim, Hye Seon Lee, Wantae Kim, Ji Hye Shin, Hyunmin Kim, Ho-Chul Shin, Hwangseo Park, Byung-Ha Oh, Won Kon Kim, Kwang-Hee Bae, Sang Chul Lee, Eun-Woo Lee, Bonsu Ku, Seung Jun Kim

**Affiliations:** 1 Disease Target Structure Research Center, Korea Research Institute of Bioscience and Biotechnology, Daejeon, Republic of Korea; 2 Department of Bioscience, University of Science and Technology KRIBB School, Daejeon, Republic of Korea; 3 Metabolic Regulation Research Center, Korea Research Institute of Bioscience and Biotechnology, Daejeon, Republic of Korea; 4 Department of Biology, Chungnam National University, Daejeon, Republic of Korea; 5 Rare Disease Research Center, Korea Research Institute of Bioscience and Biotechnology, Daejeon, Republic of Korea; 6 Department of Biochemistry, Chungnam National University, Daejeon, Republic of Korea; 7 Department of Biological Sciences, KAIST Institute for the Biocentury, Korea Advanced Institute of Science and Technology, Daejeon, Republic of Korea; 8 Department of Bioscience and Biotechnology, Sejong University, Seoul, Republic of Korea; University of Wisconsin-Madison, UNITED STATES

## Abstract

Human papillomaviruses (HPVs) are causative agents of various diseases associated with cellular hyperproliferation, including cervical cancer, one of the most prevalent tumors in women. E7 is one of the two HPV-encoded oncoproteins and directs recruitment and subsequent degradation of tumor-suppressive proteins such as retinoblastoma protein (pRb) via its LxCxE motif. E7 also triggers tumorigenesis in a pRb-independent pathway through its C-terminal domain, which has yet been largely undetermined, with a lack of structural information in a complex form with a host protein. Herein, we present the crystal structure of the E7 C-terminal domain of HPV18 belonging to the high-risk HPV genotypes bound to the catalytic domain of human nonreceptor-type protein tyrosine phosphatase 14 (PTPN14). They interact directly and potently with each other, with a dissociation constant of 18.2 nM. Ensuing structural analysis revealed the molecular basis of the PTPN14-binding specificity of E7 over other protein tyrosine phosphatases and also led to the identification of PTPN21 as a direct interacting partner of E7. Disruption of HPV18 E7 binding to PTPN14 by structure-based mutagenesis impaired E7’s ability to promote keratinocyte proliferation and migration. Likewise, E7 binding-defective PTPN14 was resistant for degradation via proteasome, and it was much more effective than wild-type PTPN14 in attenuating the activity of downstream effectors of Hippo signaling and negatively regulating cell proliferation, migration, and invasion when examined in HPV18-positive HeLa cells. These results therefore demonstrated the significance and therapeutic potential of the intermolecular interaction between HPV E7 and host PTPN14 in HPV-mediated cell transformation and tumorigenesis.

## Abstract 2

Human papillomaviruses cause various diseases associated with cellular hyperproliferation, including cervical cancer. Structural, biochemical, and cellular analyses reveal the molecular basis and significance of the intermolecular interaction between the E7 protein of human papillomavirus 18 and the human tumor suppressor protein PTPN14.

## Introduction

Human papillomaviruses (HPVs) are small, double-stranded DNA viruses belonging to the papillomavirus family, which infect cutaneous or mucosal epithelia and induce cellular hyperproliferation [1–3]. Among the more than 200 genotypes of HPV that have been identified thus far, at least 14 genotypes are categorized to be “high risk.” They are the critical causative agents of cervical cancer, which in 2018 is the fourth most frequent cancer, with 570,000 cases and 311,000 deaths in women worldwide [4]. High-risk HPVs are also implicated in the oncogenesis of a variety of anogenital and oropharyngeal cancers [1–3]. The genome of HPV encodes nine proteins: seven functional proteins necessary for viral replication and cell transformation (E1, E2, E4–E7, and E8) and two structural proteins constituting the viral capsid (L1 and L2) [1,2]. Among these proteins, two zinc-finger proteins, E6 and E7, are usually defined as oncoproteins. Their sustained expression can cause malignant cellular transformation and immortalization [5,6]. E6 from high-risk HPVs forms a ternary complex with the key tumor suppressor p53 and the ubiquitin ligase E6AP and brings about proteasome-mediated degradation of p53 [7], which was analyzed at an atomic level through complex structure determination [8]. E7 from high-risk HPVs recognizes other important tumor suppressors such as retinoblastoma protein (pRb) and retinoblastoma-like protein 1 (also called p107) via its LxCxE motif. This motif binds the pocket domain of those host proteins as elucidated by complex structure determination [9,10] and subsequently recruits the ubiquitin ligase Cullin 2 or Cullin 3 for their degradation via the proteasome [11,12]. HPV E7 is composed of three conserved regions (CRs): CR1 at the N-terminal end; CR2, which follows CR1 and contains the LxCxE motif; and CR3 comprising the C-terminal half of the protein [6]. Unlike CR1 and CR2, which are known to be unstructured, CR3 forms a homodimeric domain structure whose monomer is composed of two α-helices and two β-strands, in which a zinc ion is coordinated [13,14]. The CR3-containing C-terminal domain of HPV E7 was reported to associate with various host proteins involved in cellular proliferation, which include transcription factors E2F1 [15] and E2F6 [16] and histone deacetylases 1 and 2 (HDAC1 and HDAC2) [17–19], and contributes to host-cell immortalization and transformation through the pRb-independent pathway(s) [20–22]. Recently, two different research groups reported substantial evidences of oncogenesis-contributing cellular association of the HPV E7 C-terminal domain with nonreceptor-type protein tyrosine phosphatase 14 (PTPN14) [23–25], consistent with systematic proteomic/screening analyses done by other groups [12,26–28].

PTPN14—also called PTPD2, PTP36, or PTP-Pez—is a member of the protein tyrosine phosphatase (PTP) protein family. In coordination with protein tyrosine kinases, PTP proteins control protein tyrosine phosphorylation, one of the key posttranslational modifications intimately associated with the regulation of various cellular signaling and biological processes [29,30]. PTPN14 is a cytosolic PTP protein and constitutes a tumor-suppressive p53–PTPN14–yes-associated protein (YAP) axis that controls the Hippo signaling pathway [31]. It is one of the key cascades regulating cellular proliferation, survival, and apoptosis and thus is directly involved in tumorigenesis [32–34]. Under the control of p53, PTPN14 interacts with the transcriptional regulator YAP, the central downstream effector of the Hippo cascade, via its PPxY motifs in the middle region. A number of recent reports commonly indicated that PTPN14 negatively regulates the YAP function by sequestration of YAP in the cytoplasm in a phosphatase activity–independent manner [35–38]. Given that YAP is known to be potently oncogenic by activating the transcription of genes responsible for cell proliferation and antiapoptotic survival signaling [39], PTPN14 functions as an important antitumor protein. Moreover, PTPN14 was reported to be involved in the suppression of tumor cell metastasis by restricting intracellular protein trafficking [40]. These publications collectively provide a rationale for why PTPN14 is targeted by HPV for degradation [23–25]. It was reported that the cellular protein level of PTPN14 is decreased by the expression of high-risk HPV E7 proteins in a ubiquitin ligase UBR4-dependent manner and that the binding between PTPN14 and HPV E7 examined by coimmunoprecipitation is indispensable for the proteasome-mediated degradation of PTPN14 [23,24]. Interaction with PTPN14 is mediated by the C-terminal domain of HPV E7 but not by CR2, indicating that the viral protein should associate with the target host protein to form a complex in a totally unidentified manner at an atomic level. Moreover, how HPV E7 recognizes PTPN14 specifically over other PTP proteins sharing high structural similarity among them has remained undetermined, with a lack of complex structural information.

In this study, we confirmed the direct and potent interaction between the two domains and determined the crystal structure of the PTP domain of human PTPN14 in a complex with the C-terminal domain of HPV18 E7. It is the first crystal structure of HPV E7’s C-terminal domain bound to a host protein, and to the best of our knowledge, it is also the first PTP domain structure bound to a viral protein. The complex structure provided a basis for in-depth analysis of the E7-binding specificity of PTP proteins, which led to not only the identification of the nonconserved PTPN14 residues critical for complex formation but also verification of PTPN21 as a direct interacting partner of HPV E7. Through structure-based mutagenesis abrogating the HPV18 E7–PTPN14 binding, we demonstrate that the intermolecular interaction is significantly associated with HPV18-mediated proteasomal degradation of PTPN14, up-regulation of Hippo signaling downstream effectors, and enhanced cell proliferation, migration, and invasion, which was verified at a cellular level using nontumorigenic keratinocytes and HPV-positive or negative cervical cancer cells.

## Results

### The PTP domain of human PTPN14 directly interacts with the C-terminal domain of HPV E7

**Fig 1:**
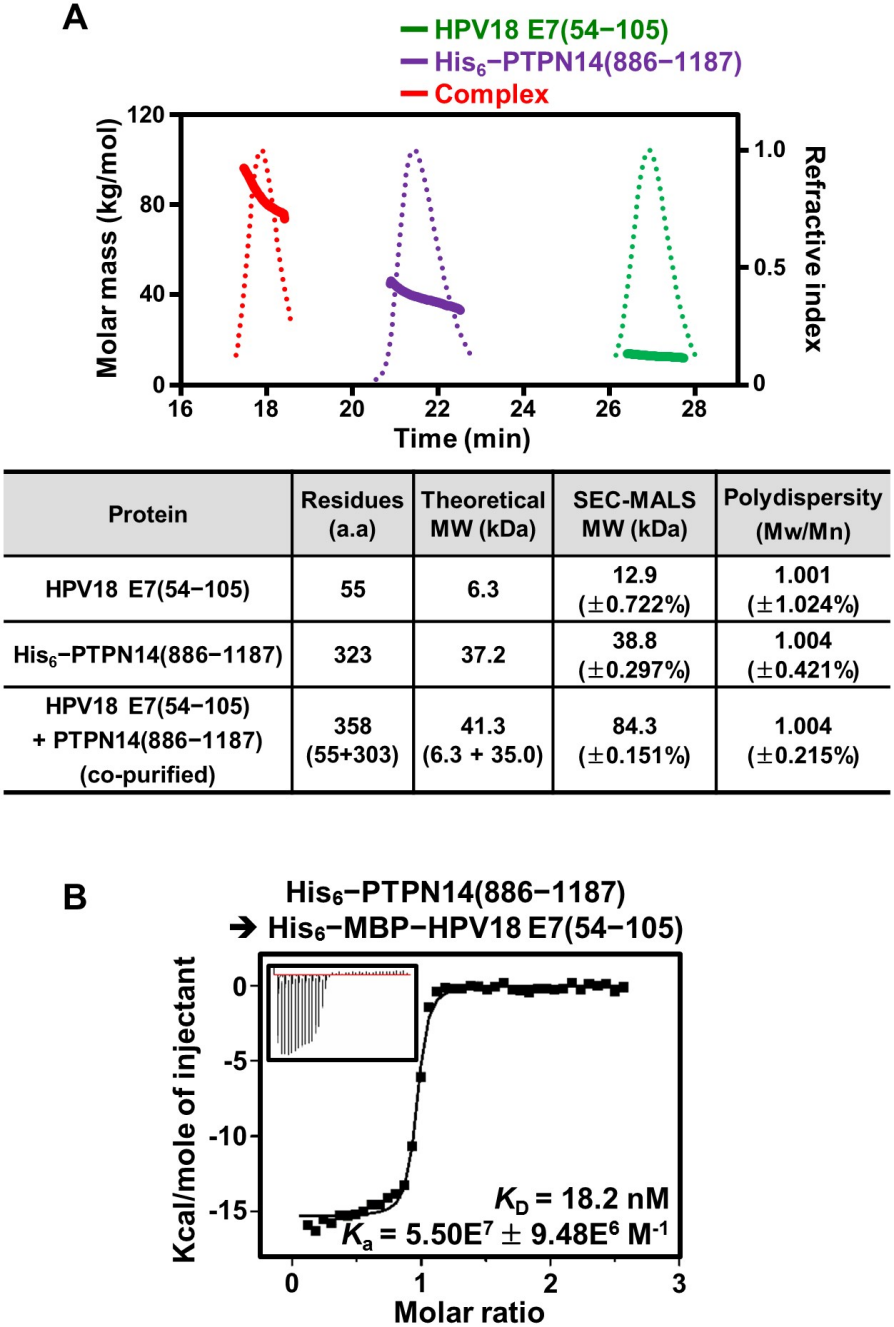
Interaction of the PTPN14 PTP domain with HPV18 E7. (A) SEC-MALS analysis. (Top) Molar masses (in kg/mol) and refractive indexes are plotted as solid and dotted lines, respectively, against the elution time (in minutes) from the size-exclusion column. (Bottom) The dimeric C-terminal domain of HPV18 E7 makes a 2:2 complex with the monomeric PTP domain of human PTPN14. Constructs are listed in S1 Table. The numerical data are included in S1 Data. (B) ITC analysis; 0.2 mM PTPN14 PTP domain was titrated into 10 μM MBP-tagged HPV18 E7. The *K*_D_ value was deduced from curve fittings of the integrated heat per mole of added ligand. a.a., amino acids; HPV, human papillomavirus; ITC, isothermal titration calorimetry; MBP, maltose binding protein; Mn, number-average molar mass; Mw, weight-average molar mass; PTP, protein tyrosine phosphatase; PTPN14, nonreceptor-type PTP 14; SEC-MALS, size-exclusion chromatography–multiangle light scattering.

Previously, several different groups reported the cellular interaction between the C-terminal domain of HPV E7 and the PTP domain of human PTPN14, which was detected by coimmunoprecipitation assays [23,24] or systematic proteomic/screening analyses [12,26–28]. We attempted to verify whether the binding between the two proteins is relevant using recombinant proteins. First, the untagged PTP domain (residues 886–1187) of human PTPN14 and the N-terminally His_6_–maltose binding protein (MBP)-tagged C-terminal domain (residues 54–105) of HPV18 E7 were separately expressed in *Escherichia coli*, after which their cell lysates were mixed for copurification. After Ni–nitrilotriacetic acid (NTA) affinity chromatography, the eluted protein sample was treated with tobacco etch virus (TEV) protease and further purified using a second round of Ni-NTA affinity chromatography to remove the His_6_–MBP tag. Finally, the protein sample was subjected to size-exclusion chromatography (SEC), and the resulting sample was assayed by sodium dodecyl sulfate (SDS)-polyacrylamide gel electrophoresis (PAGE). As shown in S1A Fig, the two proteins were eluted together, indicating that they directly interact with each other and form a tight complex. This result was further corroborated by SEC-multiangle light scattering (MALS) analysis conducted using recombinant proteins (S1 Table). Whereas the PTP domain of PTPN14 alone was measured to exist as a monomeric form in solution, the C-terminal domain of HPV18 E7 was observed to form a homodimer (Fig 1A), consistent with the previous structural elucidation that showed the homodimerization of the C-terminal domain of E7 from HPV1a or HPV45 [13,14]. Critically, when the copurified complex protein sample was subjected to SEC-MALS analysis, a single protein peak with 100% mass fraction was shown with the molecular weight calculated as a 2:2 heterodimeric form, further demonstrating the complex formation between the two proteins (Fig 1A). Finally, the intermolecular interaction between human PTPN14 and HPV18 E7 was quantified using isothermal titration calorimetry (ITC). The resulting dissociation constant (*K*_D_) of the interaction was determined to be 18.2 nM (Fig 1B), demonstrating that they interact with each other with remarkably high binding affinity.

### Structural determination of the PTPN14 PTP domain bound to the HPV18 E7 C-terminal domain

**Fig 2:**
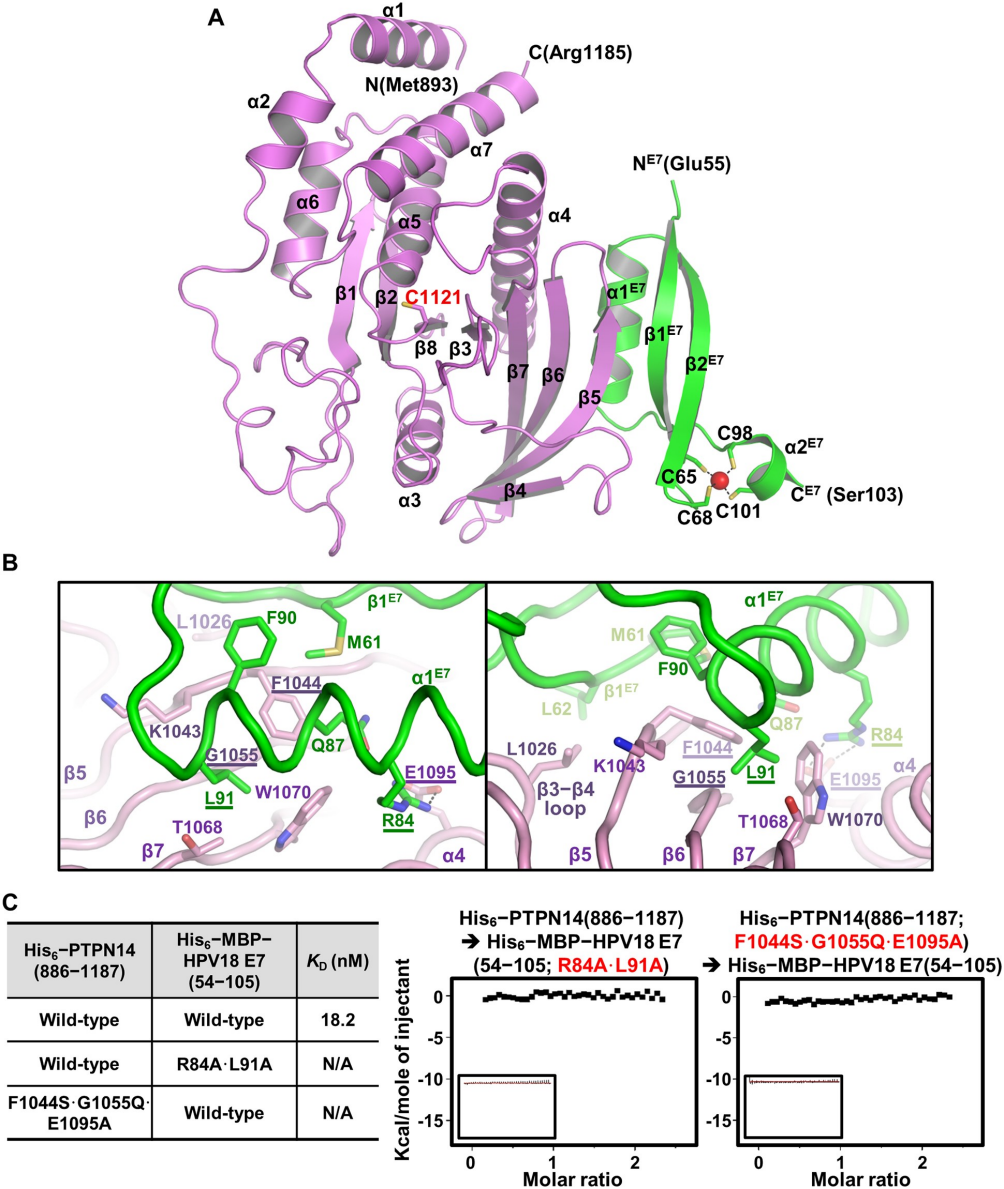
Crystal structure of the PTPN14–HPV18 E7 complex. (A) Crystal structure of the complex. PTPN14 (violet) and HPV E7 (green) are presented as ribbon drawings with the secondary structure labels according to the order of their appearance in the primary sequence. The catalytic cysteine residue of PTPN14 (Cys1121) and zinc ion (present as a red sphere)–coordinating four cysteine residues of HPV18 E7 (Cys65, Cys68, Cys98, and Cys101) are shown in sticks and labeled. (B) Intermolecular interaction is shown in two views. PTPN14 and HPV18 E7 residues critically involved in the complex formation are shown in sticks and labeled. Residues that are selected to be mutated in *c* are marked with underlines. Dotted lines represent electrostatic interaction between Glu1095 of PTPN14 and Arg84 of HPV18 E7. (C) ITC measurements. The *K*_D_ values between wild-type or mutant proteins were calculated as was in Fig 1B. Introduction of R84A and L91A mutations into HPV18 E7 or F1044S, G1055Q, and E1095A mutations into PTPN14 abrogated the binding interaction between the two proteins. HPV, human papillomavirus; ITC, isothermal titration calorimetry; MBP, maltose binding protein; PTPN14, nonreceptor-type protein tyrosine phosphatase 14.

Next, we attempted the crystallization of the PTPN14 PTP domain in a complex with the HPV18 E7 C-terminal domain, which consequently led to the determination of the complex structure to a resolution of 1.8 Å (Table 1). The PTP domain of PTPN14 contains a central twisted β-sheet composed of eight β-strands, which is surrounded by seven α-helices (Fig 2A). The interaction of HPV18 E7 does not induce a dramatic conformational change of the PTPN14 PTP domain, as its structures in the apo form and in the HPV18 E7–bound form were well matched when superimposed with a root-mean-square deviation (RMSD) value of 0.93 Å over 276 aligned residues (S2A Fig). Moreover, the conformation of main and side chains of the phosphate-binding loop containing the catalytic cysteine residue (Cys1121 of PTPN14) and the tryptophan-proline-aspartate (WPD) loop containing the general/acid base aspartate residue (Asp1079 of PTPN14), both of which are critical for the catalytic activity of the PTP protein, are not notably altered upon binding to HPV18 E7 (S2B Fig). The HPV18 E7 C-terminal domain is composed of two α-helices and two antiparallel β-strands, in which a zinc ion is captured by four cysteine residues provided from two CXXC motifs (C^65^CKC^68^ and C^98^PWC^101^) (Fig 2A). The intermolecular interactions between the two proteins are mainly mediated by α1 and β1 from the HPV18 E7 C-terminal domain and β5, β6, β7, and α4 from the PTPN14 PTP domain (Fig 2A and 2B). In detail, intermolecular hydrophobic interactions involve Phe90 and Leu91 from α1 and Met61 and Leu62 from β1 of HPV18 E7 and Lys1043 and Phe1044 from β5, Gly1055 from β6, Trp1070 from β7, and Leu1026 from the β3–β4 loop of PTPN14 (Fig 2B). Additionally, Arg84 from α1 of HPV18 E7 constitutes an electrostatic interaction with Glu1095 from α4 of PTPN14 (Fig 2B). Notably, the binding between PTPN14 and HPV18 is reinforced by a number of direct or water-mediated hydrogen bonds and by van der Waals interactions (S3 Fig).

**Table 1:** Data collection and structure refinement statistics.

Data Collection	PDB 6IWD
Space group	*P*2_1_2_1_2
Unit cell dimensions	
a, b, c (Å)	78.95, 78.95, 90.26
α, β, γ (°)	90, 90, 120
Resolution (Å)	50.0–1.8 (1.83–1.80)^a^
*R*_sym_^b^ (%)	8.7 (32.8)
*I*/σ(*I*)	41.3 (4.8)
Completeness (%)	95.1 (87.9)
Redundancy	7.6
Refinement	
Resolution (Å)	50.0–1.8
Number of reflections	35,674
*R*_work_^c^/*R*_free_ (%)	18.6/21.3
Number of atoms	
Protein	2,688
Water and ion	279
R.m.s deviations	
Bond lengths (Å)	0.007
Bond angles (°)	0.774
Ramachandran plot (%)	
Most favored region	95.8
Additionally allowed region	3.9
Outliers	0.3
Average B-values (Å^2^)	
Protein	27.4
Water and ion	34.5

^a^The numbers in parentheses are statistics from the shell with the highest resolution.

^b^*R*_sym_ = Σ |*I*_obs_—*I*_avg_| / *I*_obs_, where *I*_obs_ is the observed intensity of individual reflection and *I*_avg_ is the average across symmetry equivalents.

^c^*R*_work_ = Σ ||*F*_o_|—|*F*_c_|| / Σ |*F*_o_|, where |*F*_o_| and |*F*_c_| are the observed and calculated structure factor amplitudes, respectively. *R*_free_ was calculated with 5.61% of the data.

Abbreviations: PDB, Protein Data Bank; R.m.s., root-mean-square

Based on the complex structure, we selected Arg84 and Leu91 of HPV18 E7 and Phe1044, Gly1055, and Glu1095 of PTPN14 as key residues in the binding interaction between the two proteins. ITC measurements confirmed that alanine substitution (R84A∙L91A) of the HPV18 E7 key residues or introduction of PTP1B-mimicking mutations (F1044S∙G1055Q∙E1095A) into PTPN14 completely abrogate the binding between the two proteins (Fig 2C).

**Fig 3:**
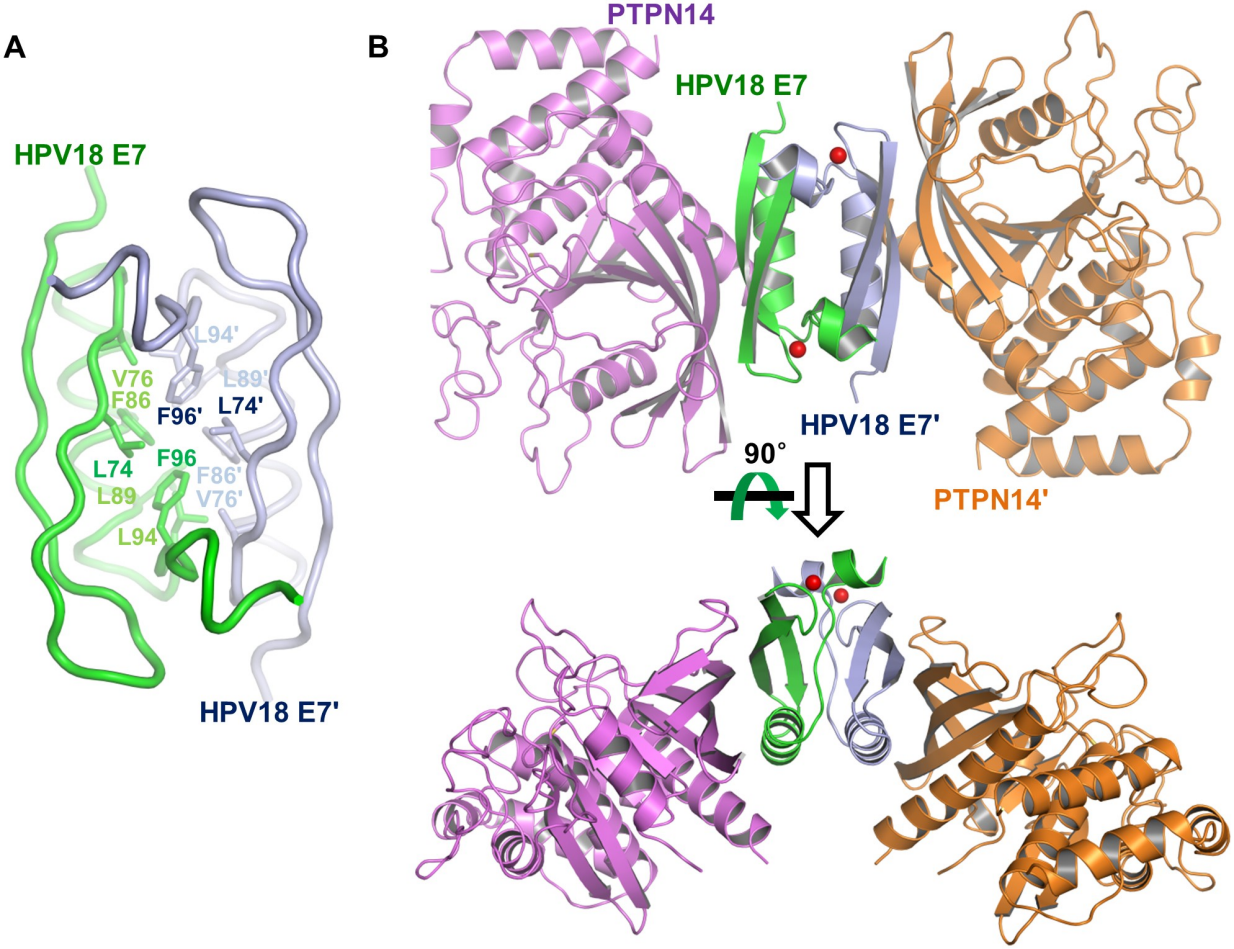
PTPN14 PTP domain and HPV18 E7 constitute a 2:2 complex. (A) C-terminal domain of HPV18 (green) forms a homodimer together with the crystallographic symmetry-associated molecule (navy). Residues involved in the intermolecular hydrophobic interaction are shown in sticks and labeled. (B) Two views of 2:2 dimeric structure. Two PTPN14–HPV18 E7 complexes are crystallographically associated with each other as shown in S4B Fig. HPV, human papillomavirus; PTPN14, nonreceptor-type protein tyrosine phosphatase 14.

Despite the fact that the asymmetric unit of our crystal contains one PTPN14 molecule bound to a single HPV18 E7 molecule, previous cellular [41–43] and structural [13,14] studies, as well as our SEC-MALS (Fig 1A) and coimmunoprecipitation analyses (S4A Fig), commonly indicated that the E7 protein of HPV forms a homodimer. The dimeric interface of HPV18 E7 was found between crystallographic symmetry-related molecules (S4B Fig). Intermolecular interaction between two E7 molecules is mediated by tight hydrophobic interaction, in which Leu74, Val76, Phe86, Leu89, Leu94, and Phe96 from each of the monomers are involved (Fig 3A). This crystallographically associated HPV18 E7 dimer overlapped greatly with the previously determined E7 structures when superimposed, with an RMSD value of 0.84 Å over 88 aligned residues of HPV1a E7 determined by X-ray crystallography, or 1.46 Å over 93 aligned residues of HPV45 E7 determined by nuclear magnetic resonance (S4C Fig). The dimerized HPV18 E7 C-terminal domain has double binding sites for the PTPN14 PTP domain, and therefore, they are able to constitute the 2:2 complex, in which two PTPN14 PTP domain molecules do not mutually contact (Fig 3B), consistent with the SEC-MALS analysis results (Fig 1A). These data collectively suggest that HPV18 E7 forms a homodimer through its C-terminal domain after being expressed in host cells, which subsequently recognizes and captures up to two PTPN14 proteins to be degraded.

### HPV18 E7 specifically binds to the PTP domain of PTPN14 and PTPN21

**Fig 4:**
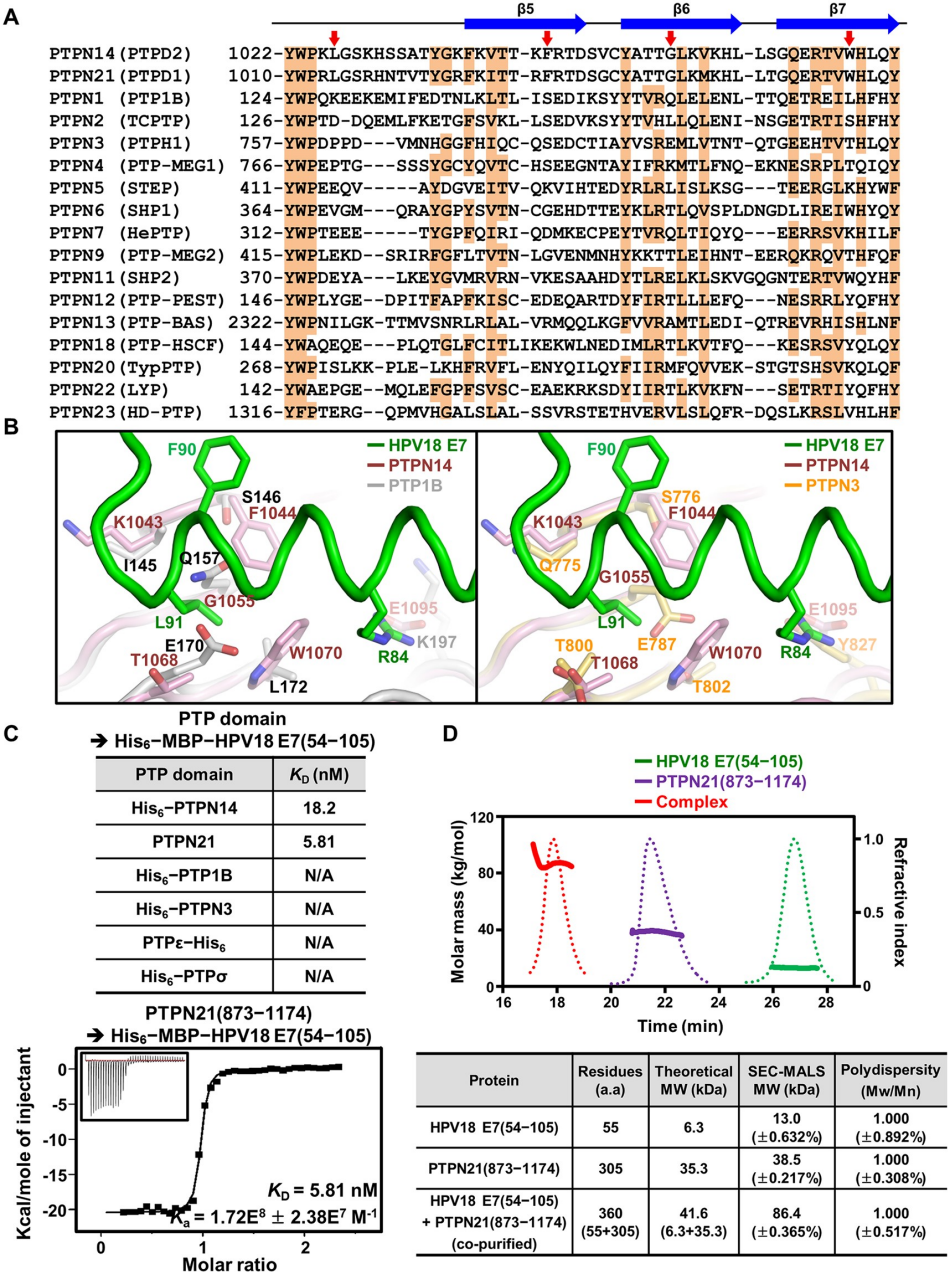
Analysis of the binding specificity of HPV18 E7 to PTP proteins. (A) Sequence alignment. Sequence of the HPV18 E7–binding interface of PTPN14 is aligned with those of the corresponding regions of 16 other nonreceptor-type PTP proteins. The secondary structures of PTPN14 are shown at the top. Conserved residues are shaded in orange. Four hydrophobic residues in PTPN14 that play a key role in the intermolecular interaction with HPV18 E7 are marked by red arrows. (B) Structural alignment. The PTP domains of two representative nonreceptor-type PTP proteins, PTP1B and PTPN3, are superimposed onto that of PTPN14 bound to HPV18 E7. Shown in sticks and labeled are PTPN14 residues interacting with Arg84 or Leu91 of HPV18 E7 and PTP1B and PTPN3 residues corresponding to the represented PTPN14 residues. (C) ITC measurements. The *K*_D_ values between the C-terminal domain of HPV18 E7 and a series of PTP domains were calculated, as in Fig 1B. Deducible dissociation constants were shown only when PTPN14 or PTPN21 was subjected to the experiment. (D) SEC-MALS analysis. The complex formation was shown between the dimeric C-terminal domain of HPV18 E7 and the monomeric PTP domain of human PTPN21. Solid lines, molar masses in kg/mol; dotted lines, refractive indexes. Constructs are listed in S1 Table. The numerical data are included in S1 Data. a.a., amino acids; HPV, human papillomavirus; ITC, isothermal titration calorimetry; MBP, maltose binding protein; Mn, number-average molar mass; Mw, weight-average molar mass; PTP, protein tyrosine phosphatase; PTPN14, nonreceptor-type PTP 14; SEC-MALS, size-exclusion chromatography–multiangle light scattering.

Classical PTP proteins, including receptor and nonreceptor types, share considerable sequence and structural homology among their catalytic PTP domain [44]. This raises the question of whether the E7 molecule interacts specifically with the PTP domain of PTPN14. To verify this issue, the amino acid sequence of PTPN14 was aligned with those of 16 nonreceptor-type PTP proteins (Fig 4A) or 20 receptor-type PTP proteins (S5 Fig). Intriguingly, we found that the key hydrophobic residues of PTPN14 that are critical for binding HPV18 E7—such as Leu1026 from the β3–β4 loop, Phe1044 from β5, Gly1055 from β6, and Trp1070 from β7—are identically conserved in PTPN21 (also called PTPD1), whose interaction with HPV E7 was previously detected by systematic proteomic analyses [12,26,27], but not in other classical PTP proteins (Fig 4A and S5 Fig). Next, we superimposed HPV18 E7–bound PTPN14 onto two representative nonreceptor-type PTP proteins, PTP1B and PTPN3, and analyzed intermolecular contacts between them. PTP1B and PTPN3 were aligned well to PTPN14, with RMSD values of 1.27 Å over 228 aligned residues and 0.70 Å over 208 aligned residues, respectively, indicating that these nonreceptor-type PTP proteins share considerable overall structural similarity between them. However, at an atomic level, we found that the E7-binding interface of PTPN14 is quite different from the corresponding region of PTP1B or PTPN3 (Fig 4B). First, the aromatic bulky hydrophobic residues (Phe1044 and Trp1070 in PTPN14) critical for the accommodation of Leu91 of HPV18 E7 are absent in PTP1B or PTPN3. Second, Leu91 of HPV18 E7 causes remarkable steric hindrance with Gln157 and Glu170 of PTP1B or with Glu787 of PTPN3 but not with the corresponding residues of PTPN14. Third, PTP1B and PTPN3 do not contain a negatively charged residue that can interact with Arg84 of HPV18 E7. Subsequent structural alignments of HPV18 E7–bound PTPN14 with four additional nonreceptor-type PTPs (PTPN7, PTPN9, SHP1, and PTPN12), as well as with two receptor-type PTPs (PTPσ and PTPε), led to the same conclusion; these PTP proteins are unable to interact with the E7 molecule because of steric hindrance with Leu91 of HPV18 E7, in addition to the differences in residues constituting the binding interface (S6 Fig). Collectively, these results suggest that HPV18 E7 specifically interacts with PTPN14, and presumably PTPN21, but not with other classical PTP proteins.

This hypothesis was validated by using ITC to measure the binding affinities between HPV18 E7 and PTP proteins including PTPN21, PTP1B, PTPN3, PTPε, and PTPσ. Indeed, it was confirmed that the C-terminal domain of HPV18 E7 interacts with the PTP domain of PTPN21 very potently, with a dissociation constant of 5.81 nM, but not with the other PTP domains (Fig 4C). SEC-MALS was also carried out using recombinant proteins. When the final purified sample (S1B Fig) was subjected to SEC-MALS, the PTP domain of PTPN21 was eluted together with the C-terminal domain of HPV18 E7 with a molecular weight of approximately 86 kDa, which is assumed to be a 2:2 complex (Fig 4D), as was that of PTPN14 (Fig 1A). Collectively, these structural and biochemical analyses provide indication of PTPN21 being a direct binding partner of HPV18 E7 with single-nanomolar binding affinity.

### PTPN14 interacts with E7 from diverse HPV genotypes

In the previous reports showing PTPN14–HPV E7 association through immunoprecipitation, it was found that PTPN14 interacts not only with E7 from high-risk HPVs, such as HPV16 and HPV18, but also with the homologues from low-risk HPVs including HPV1a, HPV4, HPV11, and HPV38 [23,24,28]. This implies that E7 from a wide range of HPV genotypes could bind PTPN14. For analyzing such an interaction profile at a molecular level, we first aligned the amino acid residues of E7’s C-terminal domain from the six HPVs for comparison. The key residues of HPV18 E7 for binding PTPN14 in the complex structure are shown to be most conserved in the other five E7 proteins (S7A Fig), suggesting that they all might interact with the PTP domain of PTPN14. Next, we attempted to confirm the complex formation in vitro using recombinant proteins. Our initial trials of measuring binding affinities using ITC were unsuccessful because of severe precipitation of several E7 proteins after being purified. When the E7 proteins were treated with TEV protease to cleave the His_6_–MBP tag for further purification, we noticed that only the E7 C-terminal domain from HPV18, but not those from other genotypes we tested, was able to be purified to homogeneity (S8 Fig). Therefore, instead of using ITC, the E7 proteins were copurified with PTPN14 by mixing their cell lysates (S1C Fig) and then subjected to SEC-MALS. As shown in S7B Fig, all of the six E7 C-terminal domains were revealed to constitute a stable complex with the PTP domain of PTPN14 with the molecular weight corresponding to a 2:2 complex (80–85 kDa). Such results demonstrate that the PTPN14-binding ability is promiscuous in HPV E7 proteins, irrespective of the virus genotypes.

### Proteasomal degradation of PTPN14 requires HPV18 E7 binding

**Fig 5:**
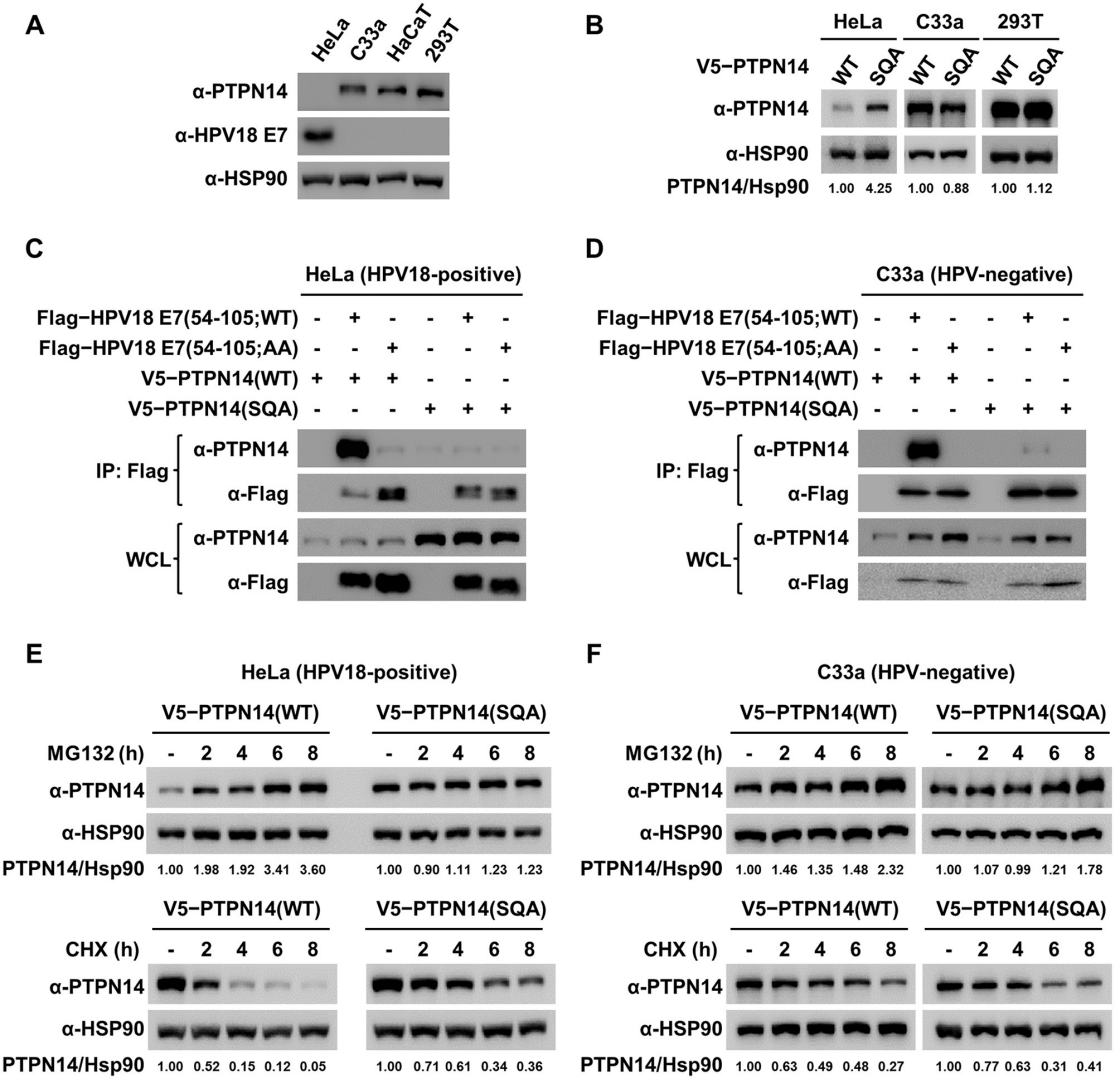
Interaction with E7 is critical for the proteasomal degradation of PTPN14. Lysates of cells transiently expressing the indicated proteins for 24 hours were subjected to IP and immunoblotting, with antibodies as marked. (A) Endogenous expressions of HPV18 E7 and PTPN14 in four cell types were verified. (B) Protein levels of PTPN14 transiently expressed in three cell types were quantified using Vilber Lourmat software with normalization to Hsp90. (C–D) Coimmunoprecipitation assay using WT and binding-defective mutant PTPN14 and HPV18 E7 C-terminal constructs in HeLa (C) or C33a (D) cells. (E–F) MG132 (top) or CHX (bottom) treatment. PTPN14 protein levels at the indicated time post 20 μM MG132 treatment for blocking proteasomal degradation (top) or 50 μg/mL CHX treatment for preventing protein synthesis (bottom) in HeLa (E) or C33a (F) cells were determined by immunoblotting. Relative protein amounts were quantified using Vilber Lourmat software and normalized to Hsp90. AA, R84A and L91A; CHX, cycloheximide; HPV, human papillomavirus; Hsp90, heat shock protein 90; IP, immunoprecipitation; PTPN14, nonreceptor-type protein tyrosine phosphatase 14; SQA, F1044S, G1055Q, and E1095A; WCL, whole-cell lysate; WT, wild type.

Through structural analysis, the mutations disrupting the complex formation between HPV18 E7 (R84A and L91A [AA]) and PTPN14 (F1044S, G1055Q, and E1095A [SQA]) were identified (Fig 2B and 2C). We attempted to elucidate whether the crystallographically and biochemically defined intermolecular interaction is physiologically relevant. To begin with, we examined the protein levels of endogenous PTPN14 and HPV18 E7 in four cell types: HPV18-positive HeLa cervical cancer cells, HPV-negative C33a cervical cancer cells, nontumorigenic HaCaT kerationocytes, and 293T cells. HPV18 E7 was verified to be expressed endogenously only in HeLa cells, which was accompanied by an undetectable protein level of endogenous PTPN14 (Fig 5A). Next, we compared the protein levels of wild-type and the SQA mutant PTPN14 constructs transiently expressed in HeLa, C33a, and 293T cells. As shown in Fig 5B, the amounts of the two PTPN14 proteins were comparable in C33a and 293T cells but not in HeLa cells; the protein level of wild-type PTPN14 was quite lower than that of the SQA mutant PTPN14 in these HPV18 E7–positive cells. Therefore, we hypothesized that wild-type PTPN14 was easily recognized by HPV18 E7 for degradation, whereas the SQA mutant PTPN14 was resistant to be degraded in HeLa cells because of the absence of key residues essential for binding HPV18 E7 (Fig 2C). To validate our hypothesis, we first performed immunoprecipitation analysis to verify protein interaction between PTPN14 and HPV18 E7 in HPV18-positive HeLa and HPV-negative C33a cells by using wild-type and mutant constructs containing the aforementioned substitutions. To avoid unwanted protein degradation and to detect the complex formation clearly in cells, the C-terminal domain constructs of HPV18 E7 were used in this step. We demonstrated that intermolecular interaction between PTPN14 and the C-terminal domain of HPV18 E7, which was detected in both HeLa (Fig 5C, column 2) and C33a (Fig 5D, column 2) cells, was evidently abrogated by the introduction of binding-defective mutations (Fig 5C and 5D, column 3–6). Moreover, consistently with Fig 5B, the protein level of the HPV18 E7 binding-defective mutant PTPN14 was substantially higher than that of wild-type PTPN14 in HeLa cells (Fig 5C, whole-cell lysate [WCL]; α-PTPN14 lane) but not in C33a cells (Fig 5D, WCL; α-PTPN14 lane). As the two PTPN14 proteins were verified to be similar in their protein stability via a thermal shift assay performed using recombinant proteins (S9 Fig), we assumed that HPV18 E7–mediated proteasomal degradation might be the reason causing such a protein-level difference only shown in HPV18-positive cells. To address this issue, HeLa and C33a cells were treated with a proteasome inhibitor MG132 or a protein synthesis inhibitor cycloheximide (CHX), and protein accumulation and degradation of PTPN14 were analyzed at different time points. Upon MG132 treatment, a time-dependent accumulation of PTPN14 was shown in HeLa cells expressing wild-type PTPN14 to a remarkable degree but not in cells expressing the SQA mutant form (Fig 5E, top). This indicated that the HPV18 E7 binding-defective mutations protect PTPN14 from proteasomal degradation. In contrast, MG132 treatment did not affect the protein level of either wild-type or the SQA mutant PTPN14 in HPV-free C33a (Fig 5F, top) or 293T cells (S10A Fig). Consistently, when protein synthesis was blocked by CHX treatment, the protein level of wild-type PTPN14 declined much more rapidly compared to that of the HPV18 E7 binding-defective mutant PTPN14 in HeLa cells (Fig 5E, bottom) but not in C33a cells (Fig 5F, bottom). Collectively, these data demonstrate that the structurally and biochemically determined intermolecular interaction between PTPN14 and HPV18 E7 is physiologically relevant and that the HPV18 E7–mediated proteasomal degradation of PTPN14 depends on direct interaction between the two proteins.

### PTPN14 binding-defective mutations attenuate HPV18 E7’s ability to induce keratinocyte proliferation and migration

**Fig 6:**
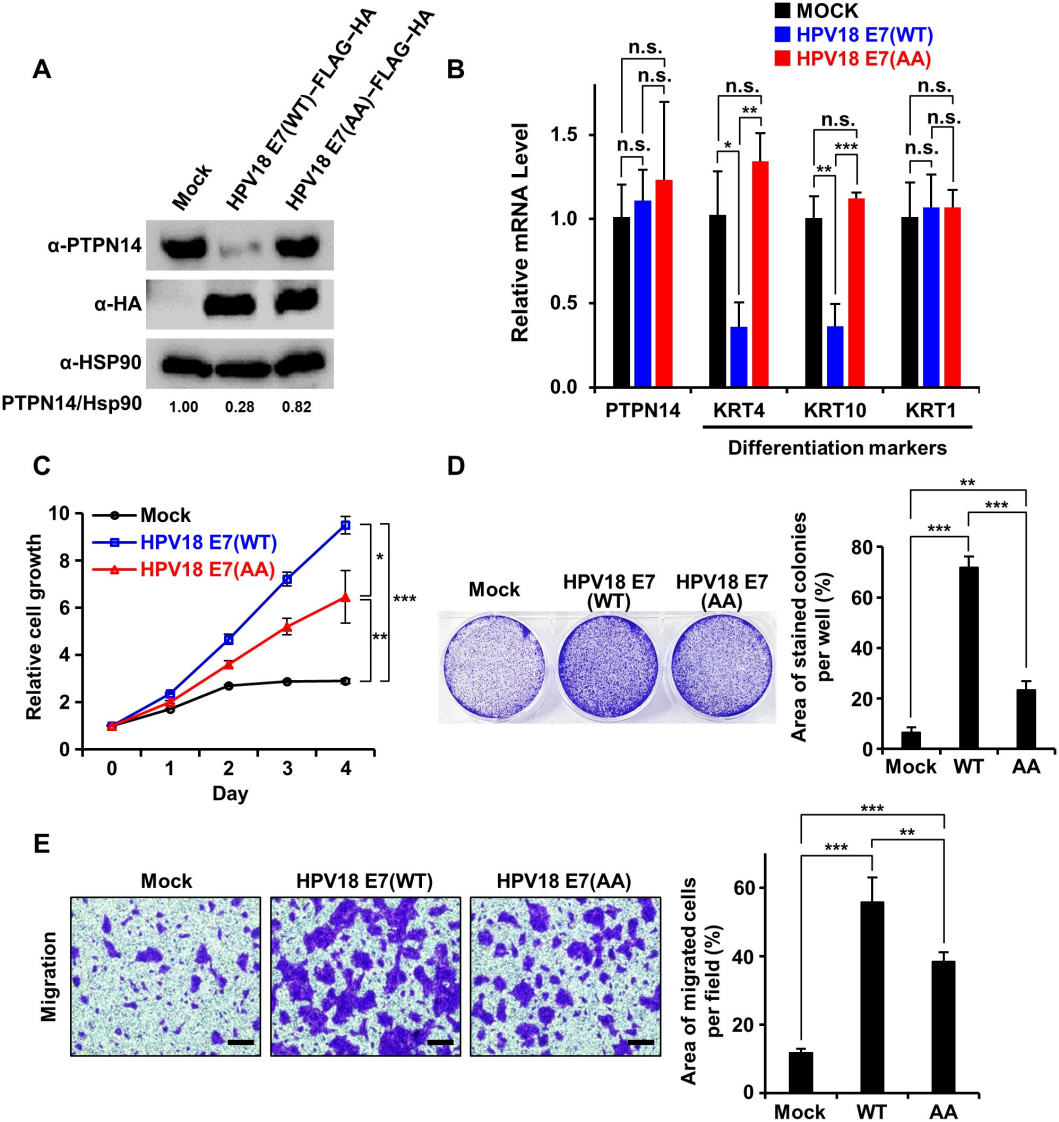
HPV18 E7 relies on interaction with PTPN14 to promote keratinocyte proliferation and migration. The indicated HPV18 E7 constructs were stably expressed in HaCaT cells. *P < 0.05; **P < 0.01; ***P < 0.001 in the Student *t* test. The numerical data are included in S1 Data. (A) Protein levels of endogenous PTPN14 upon expression of the indicated E7 constructs were detected by immunoblotting. Relative protein amounts were quantified using Vilber Lourmat. (B) mRNA levels of PTPN14 and three indicated keratinocyte differentiation markers with or without WT or the AA mutant HPV18 E7 were measured by qRT-PCR. (C) Proliferation assay. Growth curves of HaCaT cells stably expressing the indicated constructs were compared from day 0 to day 4. (D) Clonogenic assay using HaCaT cells grown for 6 days. (Left) Representative cell images stained with crystal violet. (Right) Crystal violet-stained colony area per well was quantified and shown as a bar graph. (E) Migration assay. Motility of HaCaT cells with or without WT or the AA mutant HPV18 E7 were analyzed and compared. (Left) Representative cell images stained with crystal violet. The scale bars indicate 50 μm. (Right) The area of migrated cells per field was displayed as bar graphs. AA, R84A and L91A; HA, hemagglutinin; HPV, human papillomavirus; Hsp90, heat shock protein 90; KRT, keratin; n.s., not significant; PTPN14, nonreceptor-type protein tyrosine phosphatase 14; qRT-PCR, quantitative real-time polymerase chain reaction; WT, wild type.

Having known that human keratinocytes are the targets of HPV infection causing oncogenesis, the necessity of HPV18 E7 binding to PTPN14 in nontumorigenic keratinocytes was our next issue to be addressed. For this purpose, we transduced HaCaT cells with retroviruses encoding wild-type or the PTPN14 binding-defective AA mutant HPV18 E7 and with empty-vector control. The protein level of endogenous PTPN14 was confirmed to be significantly reduced in keratinocyte cell lines stably expressing wild-type HPV18 E7 but not in those expressing the AA mutant HPV18 E7 (Fig 6A), further supporting that HPV18 E7–induced degradation of PTPN14 relies on intermolecular binding between the two proteins. A recent report also indicated that E7-mediated PTPN14 degradation results in an impairment of keratinocyte differentiation that plays a key role in the HPV-involved oncogenesis [25]. We therefore analyzed the expression levels of three representative keratinocyte differentiation markers in our HaCaT stable cell lines. Fig 6B shows that the mRNA levels of Keratin 4 (KRT4) and KRT10 were remarkably reduced by expression of wild-type HPV18 E7 but not by the AA mutant HPV18 E7, supporting the notion that E7 controls keratinocyte differentiation as previously reported [25]. Next, we further investigated the effects of expression of wild-type or the AA mutant HPV18 E7 on the cell growth and motility of keratinocytes. We noticed that wild-type HPV18 E7 is much more effective in promoting cell growth (Fig 6C and 6D) and migration (Fig 6E) compared to the PTPN14 binding-defective mutant HPV18 E7 in stable HaCaT cell lines. Even though the AA mutant E7 construct might maintain its ability to provoke the pRB-dependent tumorigenesis pathway through its CR1 and CR2 regions, we noticed that the E7 activities in HaCaT cells were severely impaired by the PTPN14 binding-defective mutations (Fig 6C–6E), demonstrating the significance of interaction with this host tumor suppressor protein for the functionality of HPV18 E7. Collectively, these results demonstrated that HPV18 E7 up-regulates cell proliferation and mobility of keratinocytes, which is accompanied by reduction of PTPN14 in the protein level and repression of keratinocyte differentiation markers in the mRNA level. Our data also indicated that not all but a substantial portion of such activities of the viral oncoprotein HPV18 E7, which should to be associated with its oncogenic property, depend on interaction with PTPN14.

### Disruption of E7 binding to PTPN14 retards Hippo downstream signaling and HeLa cell proliferation, migration, and invasion

**Fig 7:**
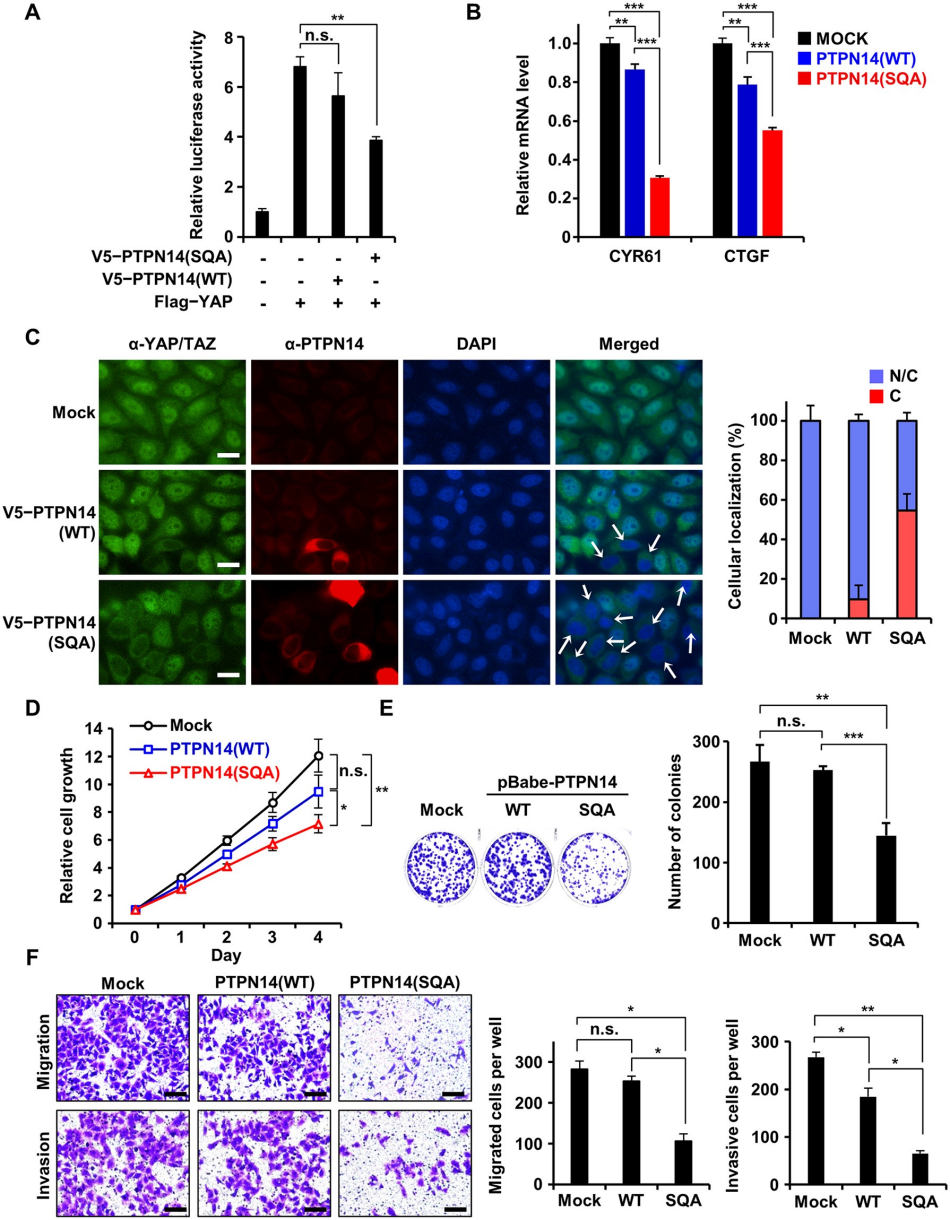
Tumor-suppressive activity of PTPN14 was recovered in HeLa cells by disrupting interaction with HPV18 E7. The indicated PTPN14 constructs were transiently (A and B) or constitutively (C–E) expressed in HeLa cells. *P < 0.05; **P < 0.01; ***P < 0.001 in the Student *t* test. The numerical data are included in S1 Data. (A) Dual luciferase assay. Transcriptional activity of YAP with or without WT or the SQA mutant PTPN14 was monitored by quantifying luciferase activity. (B) mRNA levels of CYR61 and CTGF with or without WT or the SQA mutant PTPN14 were measured by qRT-PCR and compared. (C) Subcellular localization of YAP/TAZ with or without wild-type or mutant PTPN14 was detected by immunostaining using an anti-YAP/TAZ antibody (green), anti-PTPN14 antibody (red), and DAPI (blue). Nuclear exclusion of YAP/TAZ was indicated by arrows. Percentages of YAP/TAZ located in both nucleus and cytoplasm (“N/C”) or in cytoplasm (“C”) are shown as graphs. Images were captured using 40x oil immersion objectives. The scale bars indicate 10 μm. (D) Short-term cell proliferation assay. Growth curves of HeLa cells constitutively expressing empty-vector control (“Mock”) or wild-type or the SQA mutant PTPN14 are compared from day 0 to days 4. (E) Clonogenic assay using HeLa cells grown for 15 days. (Left) Representative cell images stained with crystal violet. (Right) The number of colonies were quantified as bar graphs. (F) Migration and invasion assay. Motility and invasiveness of HeLa cells with or without wild-type or the SQA mutant PTPN14 were analyzed and compared. (Left) Representative cell images stained with crystal violet. The scale bars indicate 50 μm. (Right) The number of migrated (top) or invasive (bottom) cells were quantified as bar graphs. CTGF, connective tissue growth factor; CYR61, cysteine-rich angiogenic inducer 61; HPV, human papillomavirus; n.s., not significant; PTPN14, nonreceptor-type protein tyrosine phosphatase 14; qRT-PCR, quantitative real-time polymerase chain reaction; SQA, F1044S, G1055Q, and E1095A; TAZ, tafazzin; WT, wild type; YAP, yes-associated protein.

In high-risk HPV-infected cervical cancer cells, the protein level of endogenous PTPN14 was reported to be very low because of E7-mediated proteasomal degradation [23,24], consistent with our results (Fig 5A). Herein, we examined whether reintroduction of the SQA mutant PTPN14, confirmed to be resistant to E7-associated degradation, could restore the tumor-suppressive function of PTPN14 in HeLa cells. Recent studies revealed Hippo signaling as the major target of PTPN14 for tumor suppression, which mainly relies on the intermolecular interaction between the PPxY domain of PTPN14 and YAP or large tumor suppressor 1 (LATS1), key Hippo signaling pathway components [35–38,45]. We first confirmed that mutation introduction into the PTPN14 PTP domain did not affect the binding ability of PTPN14 to YAP or LATS1 (S10B Fig). We then examined whether expression of the SQA mutant PTPN14 could lead to suppression of transcription activities of YAP. Dual luciferase transient transfection assay using YAP-responsive promoter demonstrated that the activity of YAP was noticeably repressed by the HPV18 E7 binding-defective mutant PTPN14 but not significantly by wild-type PTPN14 in HeLa cells (Fig 7A). Consistently, the SQA mutant PTPN14 was much more effective in down-regulating endogenous mRNA levels of cysteine-rich angiogenic inducer 61 (CYR61) and connective tissue growth factor (CTGF), two bona fide YAP target genes [46], compared to wild-type PTPN14 (Fig 7B). We assumed that these are due to the difference in the amount of PTPN14 affected by E7-mediated proteasomal degradation in HeLa cells. Contrastively, when the same assay was carried out using 293T or C33a cells that do not harbor HPV18 E7 (Fig 5A), no remarkable difference in YAP activity inhibition was shown between cells expressing wild-type and the SQA mutant PTPN14 (S10C and S11A Figs). It further supported our hypothesis that the activation of the Hippo downstream pathway is determined by the level of PTPN14 protein. PTPN14 is known to prevent YAP and its functionally redundant protein tafazzin (TAZ) from translocating into the nucleus, where YAP and TAZ promote transcription associated with cell cycle progression [36,38,45]. We therefore evaluated the effect of wild-type or the SQA mutant PTPN14 expression on the subcellular localization of YAP/TAZ (Fig 7C). Endogenous YAP/TAZ primarily localized in nucleus of HeLa cells, which was hardly affected by transient expression of wild-type PTPN14. In contrast, upon expression of the SQA mutant PTPN14, a large portion of YAP/TAZ was situated in the cytosol, repressing its activity as a transcriptional coactivator. These results indicate that the activity of downstream effectors of the Hippo signaling pathway is up-regulated in cervical cancer cells by E7-mediated degradation of PTPN14, which could be retarded by interfering with the interaction between host PTPN14 and viral E7 proteins.

Given that Hippo signaling is directly associated with cell proliferation, migration, and invasion, we suspect that degradation of PTPN14 might be one of the key processes implicated in cellular transformation and tumorigenesis. To assess this hypothesis, we established stable HeLa cell lines constitutively expressing empty vector (mock), wild-type PTPN14, or the SQA mutant PTPN14 and investigated the effects of PTPN14 expression on cervical cancer cells. Fig 7D shows that HeLa cell growth was moderately suppressed by wild-type PTPN14 but considerably reduced by the SQA mutant PTPN14 in a short-term proliferation assay. In a clonogenic assay, the number of colonies was significantly reduced in the SQA mutant PTPN14-expressing HeLa cells compared to those in control or wild-type PTPN14-expressing HeLa cells (Fig 7E). Subsequently, we examined the effects of wild-type and the SQA mutant PTPN14 expression on the motility and invasiveness of HeLa cells. As shown in Fig 7F, constitutive expression of the SQA mutant PTPN14, but not that of wild-type PTPN14 or empty vector, caused a considerable suppression of migration and invasion of HeLa cells. In contrast, in HPV-negative C33a cells, wild-type PTPN14 was as effective as the SQA mutant form in suppressing cell proliferation (S11B Fig) and migration (S11C Fig), reflecting their comparable cellular protein levels (Fig 5B). Taken together, removal of tumor-suppressive PTPN14 by E7-mediated degradation should be responsible for the enhanced cell proliferation, migration, and invasion of HPV18-infected cells, which could be inhibited by blocking their complex formation.

## Discussion

E6 and E7 proteins are two defined oncoproteins encoded by HPV that are critically responsible for immortalization and transformation of HPV-infected human cells. Therefore, they have been under intensive investigation in order to understand their functions and molecular mechanisms. In this study, we verified the direct and potent molecular interaction of the C-terminal domain of HPV18 E7 and the PTP domain of human PTPN14. This was demonstrated by measuring the molecular weight of the complex by means of SEC-MALS (Fig 1A), calculating the dissociation constant between the two domains using ITC (Fig 1B), determining the complex crystal structure (Figs 2 and 3), and confirming the physiological binding in cells via coimmunoprecipitation (Fig 5C and 5D). This study provides the first atomic details of the host protein recognition by HPV E7 in the LxCxE motif-independent manner. Thus, it expands our understanding of the molecular mechanism of the noncanonical E7-mediated host protein degradation pathway, reported to be mediated by E3 ubiquitin-protein ligase UBR4/p600. We also note that our complex structure is, to the best of our knowledge, the first three-dimensional structure of the human PTP domain bound to a domain derived from a viral protein. It provides a de novo protein-binding interface for the PTP family proteins, composed of three β-strands (β5, β6, and β7) and one α-helix (α4) apart from the catalytic site (see Fig 2A).

Identification of key residues of PTPN14 (Phe1044, Gly1055, and Glu1095) and HPV18 E7 (Arg84 and Leu91) for the complex formation enabled us to design mutant E7 and PTPN14 proteins defective in binding each other. It was confirmed by ITC using recombinant proteins (Fig 2C) and coimmunoprecipitation using HeLa and C33a cells (Fig 5C and 5D). Using these mutant proteins, we demonstrated the significance of the direct interaction of E7 of HPV18, one of the two major high-risk HPVs, with PTPN14, which is necessary for inducing degradation of this host antitumor protein (Figs 5 and 6A), up-regulating the cancer-associated Hippo signaling downstream effectors (Fig 7A–7C), and promoting cell proliferation, migration, and invasion (Figs 6C–6E and 7D–7F). Simultaneously, our data clearly present a possibility that HPV-mediated cervical cancer could be suppressed by interrupting their intermolecular binding interaction. Critically, the structural and biochemical analysis revealed that the HPV18 E7–interacting selectivity for PTPN14 over other PTP proteins relies on the E7-binding site residues, which are not conserved in other receptor or nonreceptor-type PTP proteins (Fig 4, S5 and S6 Figs). This suggests that the protein–protein interaction site of PTPN14 could be a novel and suitable target for a therapeutic approach toward cervical cancer. One crucial point that should be considered is that HPV might not neutralize PTPN14 by a single route; PTPN14 was reported to be activated by p53 [31], which is the direct target of HPV E6 for degradation [8]. It therefore would be possible that E6-mediated removal of p53 leads to down-regulation of PTPN14 at an mRNA level, which might eventually contribute to HPV-associated tumorigenesis, in coordination with E7-mediated direct degradation of PTPN14. We therefore suggest that whether HPV E6 is indeed involved in PTPN14 neutralization might be an important issue to be addressed in future research. It may allow the establishment of a precise therapeutic strategy against cervical cancer.

Our structure-based sequence alignment led to the identification of PTPN21 as another direct binding partner of HPV18 E7 (Fig 4A). The potent intermolecular interaction between the PTP domain of PTPN21 and the C-terminal domain of HPV18 E7 was verified by ITC (Fig 4C) and SEC-MALS (Fig 4D). Because a previous report indicated that PTPN21 is another regulator of the Hippo pathway signaling that interferes with YAP activity, as does PTPN14 [47], it might be reasonable to hypothesize that HPV targets PTPN21 via its E7 protein during infection. It could be another intriguing issue to be explored. On the contrary, the residues of HPV18 E7 critical for the complex formation, such as Arg84 and Leu91 (Fig 2C), are well conserved in the E7 proteins from the other genotypes of HPV (S7A Fig), suggesting that all of these E7 proteins might bind PTPN14. The promiscuous PTPN14-binding property of E7 proteins derived from different HPV genotypes was validated by SEC-MALS (S7B Fig), which is also consistent with the previous data showing a similar phenotype [23,24,28]. It is notable that the previous studies also indicated that the E7 proteins from the high-risk HPVs, but not from the low-risk HPVs, were able to induce UBR4/p600-mediated PTPN14 degradation, although all the E7 proteins associated with the host protein [23,24]. Whether the interaction with E7 in the low-risk HPV infection affects the physiological role of PTPN14, presumably by disrupting its proper localization or association with host proteins, remains to be elucidated. Whether PTPN14 is targeted by other carcinoviruses might be another issue to be addressed.

### Concluding remark

In this study, we provided the structural and molecular basis for how HPV recognizes human PTPN14 to be degraded via its E7 protein. They were subsequently proven to be relevant in various aspects of the HPV18-mediated cervical cancer cell physiology. Our structural analysis also identified human PTPN21 as a direct binding partner of HPV18 E7, which was biochemically verified. Our work therefore expands our understanding of the pRB-independent tumorigenesis pathway mediated by the HPV-encoded oncoprotein E7. We also expect that our observations would be rational grounds for therapeutic approaches toward HPV-implicated malignant diseases.

## Materials and methods

### Preparation, crystallization, and structural determination of PTPN14 in a complex with HPV18 E7

The DNA fragment coding for the PTP domain of human PTPN14 (residues 886–1187) was cloned into the pET21a plasmid (Novagen). The DNA fragment coding for the C-terminal domain of HPV18 E7 (residues 54–105) was cloned into the pET28a plasmid (Novagen) that was modified to tag the protein with N-terminal (His)_6_-linked MBP. Recombinant proteins were separately produced in the *E*. *coli* BL21(DE3) RIL strain (Novagen) cultured in 2 L (for PTPN14) or 4 L (for HPV18 E7) Luria-Bertani media at 25 °C for 16 hours. After centrifugation at 1,800*g* for 30 minutes at 4 °C, each of the cells expressing PTPN14 or HPV18 E7 were resuspended in a lysis buffer containing 50 mM Tris-HCl (pH 7.5), 500 mM NaCl, 10% glycerol, and 5 mM βME and then mixed for copurification as the complex form. After sonication on ice, cell debris was removed by centrifugation of the cell lysate at 19,000*g* for 50 minutes at 4 °C. The supernatant was loaded onto a Ni-NTA column (QIAGEN) for initial purification. About 250 mg of the PTPN14–HPV18 E7 protein complex was eluted with 50 mL buffer containing 50 mM Tris (pH 7.5), 200 mM NaCl, 3 mM βME, and 300 mM imidazole. After the removal of the N-terminal tag from HPV18 E7 through 3 mg TEV protease treatment at 4 °C for 16 hours, proteins were subjected to a second Ni-NTA column and equilibrated with 50 mL buffer containing 50 mM Tris (pH 7.5), 50 mM NaCl, 3 mM βME, and 50 mM imidazole. Eluted samples were then subjected to a HiTrap Q anion exchange column (GE Healthcare) with a gradient from 0 to 300 mM NaCl in a buffer solution containing 50 mM Tris (pH 7.5) and 3 mM βME. Recombinant proteins were further purified using a HiLoad 26/600 Superdex 75 pg gel filtration column (GE Healthcare) and equilibrated with a buffer solution containing 50 mM Tris-HCl (pH 7.5), 200 mM NaCl, and 2 mM dithiothreitol. Crystals were obtained by the sitting-drop vapor-diffusion method at 18 °C by mixing and equilibrating 1 μL protein solution (10 mg/mL) and 1 μL precipitant solution containing 0.1 M Tris-HCl (pH 8.0), 25% (w/v) polyethylene glycol 3350, 0.25 M lithium sulfate monohydrate, and 2% (v/v) *tert*-butanol. Before the data collection process, crystals were immersed briefly in a cryoprotectant solution, which was a reservoir solution containing 10% glycerol. Diffraction data were collected on the beamline 5C at the Pohang Accelerator Laboratory, Korea, and processed using the program *HKL*2000 [48]. The complex structure was determined by molecular replacement method with the program Phaser [49], using the apo structure of the PTPN14 PTP domain (Protein Data Bank code 2BZL) as a search model. The programs Coot [50] and PHENIX [51] were used for model building and refinement, respectively. The final model does not include residues 886–892, 1109–1112, and 1186–1187 of PTPN14 and residues 54 and 104–105 of HPV18 E7, because their electron densities were not observed or very weak. The final model contains 19 residues (17 from PTPN14 and 2 from HPV18 E7) whose real-space R-factor z-score is higher than 2, indicating their poor fit to the electron density. We note that most of them are located in surface-exposed flexible loop regions. Crystallographic data statistics are summarized in Table 1. Constructs of recombinant proteins prepared for crystallization are shown in S2 Table.

### Preparation of recombinant proteins

The PTP domain of PTPN14 containing the F1044S∙G1055Q∙E1095A mutation and the C-terminal domain of HPV18 E7 containing R84A∙L91A mutation were prepared and purified in a manner similar to that for the wild-type proteins. Each of the PTP domains of PTP1B (residues 1–321), PTPN3 (residues 628–913), and PTPσ (residues 1367–1948) were cloned into the pET28a plasmid, and that of PTPε (residues 107–398) was cloned into the pET21a plasmid (Novagen). These four PTP proteins were purified using a Ni-NTA column and dialyzed against a buffer containing 50 mM Tris-HCl (pH 7.5), 200 mM NaCl, and 10 mM β-mercaptoethanol. The PTP domain of PTPN21 (residues 873–1174) was cloned into the pPRoEX HTa plasmid (Invitrogen) that was modified to tag the protein with N-terminal (His)_10_-linked green fluorescent protein. The PTPN21 PTP domain was purified using a Ni-NTA column and subjected to TEV protease treatment and subsequent secondary Ni-NTA column purification. Each of the C-terminal domains of E7 from HPV1a (residues 39–93), HPV4 (residues 44–100), HPV11 (residues 45–98), HPV16 (residues 45–98), and HPV38 (residues 45–100) were cloned into the MBP-coding gene-inserted modified pET28a plasmid and purified similarly with that of E7 from HPV18.

### SEC-MALS

Protein samples were diluted to a concentration of 6 mg/mL for the HPV E7 C-terminal domain or 3 mg/mL for the PTP domain and for the complex in a buffer containing 50 mM Tris-HCl (pH 7.5), 200 mM NaCl, and 2 mM dithiothreitol. SEC-MALS was carried out using a Superdex 75 10/300 GL column (GE Healthcare). The differential refractive index spectra were obtained using Optilab T-rEX (Wyatt Technology), which was combined with high-performance liquid chromatography (Shimadzu) and DAWN HELEOS-II (Wyatt Technology). The weight-average molar mass was calculated using ASTRA 6 software (Wyatt Technology).

### Isothermal titration calorimetry

All measurements were performed at 25 °C on a VP-ITC microcalorimetry system (MicroCal). Protein samples were dialyzed against the solution containing 20 mM Tris-HCl (pH 7.5), 100 mM NaCl, and 10 mM β-mercaptoethanol. In all experiments, PTP was titrated into HPV E7, in which a monomeric PTPN14 molecule interacts with a single HPV18 E7 molecule in a one-to-one binding mode. Prior to the measurements, the samples were centrifuged to remove any residuals. Dilution enthalpies were measured in separate experiments (titrant into buffer) and subtracted from the enthalpies of the binding between the protein and the titrant. Data were analyzed using the Origin software (OriginLab) as the one-to-one interaction mode. All experiments were performed in triplicate.

### Plasmid preparation for cellular assays

Plasmids p6641 MSCV-P C-FlagHA 18E7-Kozak (#35019; deposited by Prof. Peter Howley) [12] and pcDNA3-V5-PTPN14-wild type (#61003; deposited by Prof. Jianmin Zhang) [37] were obtained from Addgene and used for transient transfection and also as templates for following construct preparation. The DNA fragment coding for the C-terminal domain of HPV18 E7 was subcloned into the pcDNA3-Flag plasmid (Addgene). It was used as a template for the site-directed mutagenesis to generate a R84A and L91A double mutant construct, using a KOD-Plus-Mutagenesis kit (Toyobo). The full-length HPV18 E7 construct containing R84A∙L91A mutations and the PTPN14 construct containing F1044S∙G1055Q∙E1095A mutations were generated similarly. The pBabe-puro PTPN14 wild-type and the SQA mutant constructs were generated by inserting the DNA fragments into the pBabe-puro plasmid (Addgene) using the EcoRI site. Flag-tagged YAP and LATS1 were prepared as previously described [52,53]. Constructs used in cellular assays are listed in S3 Table.

### Cell culture, transfection, and retroviral infection

HeLa, 293T, and HaCaT cells were maintained in Dulbecco’s modified Eagle’s medium (DMEM; Corning) supplemented with 10% fetal bovine serum (GE Healthcare) and 1% antibiotic-antimycotic (GIBCO) at 37 °C, 5% CO_2_. C33a cells were purchased from ATCC and cultured in Eagle’s Minimum Essential Medium (ATCC) supplemented with 10% fetal bovine serum and 1% antibiotic-antimycotic at 37 °C, 5% CO_2_. Transient transfection of plasmids into 293T, HeLa, and C33a cells was carried out using polyethylenimine (Sigma-Aldrich) and Lipofectamine 3000 (Invitrogen), respectively, according to the manufacturer’s instructions. For establishment of stable cell lines, retroviral supernatants were generated by transfection of 293T cells with target plasmids and viral packaging plasmids VSV-G and GAG-pol (Addgene) using Lipofectamine 3000 for 48 hours. Supernatants were filtered using a 0.45-μm filter and transferred into HeLa and HaCaT cells in the presence of 8 μg/mL polybrene. Infected cells were selected with 1–2 μg/mL puromycin for 7 days.

### Immunoblotting and immunoprecipitation

For western blot analyses, cells were resuspended in phosphate-buffered saline (PBS). Samples were mixed with the 2X SDS sample buffer in a 1:1 ratio and boiled at 4 °C for 10 minutes. For immunoprecipitation analysis, cells were lysed in a buffer containing 50 mM Tris-HCl (pH 7.5), 150 mM NaCl, 0.5% Triton X-100, 1 mM ethylenediaminetetraacetic acid, and a protease inhibitor cocktail (Roche). The cell lysates were subjected to sequential immunoprecipitation using anti-Flag M2 affinity gel (A2220; Sigma) overnight at 4 °C and Protein G-Sepharose (GE Healthcare) for 2 hours at 4 °C. The immunoprecipitates were then mixed with 2X SDS sample buffer and boiled for 5 minutes. Proteins were separated by SDS-PAGE and transferred onto a 0.2-μm nitrocellulose membrane (GE Healthcare). The membranes were blocked with 5% nonfat dry milk in Tris-buffered saline with Tween 20 for 1 hour and probed with the appropriate primary antibodies for overnight at 4 °C. The membranes were then washed with Tris-buffered saline with Tween 20 three times and probed with appropriate HRP-conjugated secondary antibodies (sc-2357 and sc-516102; Santa Cruz Biotechnology). Proteins were detected using ECL reagents (Amersham) according to the manufacturer’s instructions. Antibody-bound proteins on the membrane were detected with ChemiDoc (Vilber Lourmat). The following antibodies were used for immunoblotting: anti-PTPN14 (#13808; Cell signaling), anti-HSP90 (SC-7947; Santa Cruz), anti-Flag (F3165; Sigma-Aldrich), anti-HA (12013819001; Sigma), anti-HPV18 E7 (ab100953; Abcam), and anti-GAPDH (ab9485; Abcam). MG132 and CHX were purchased from AG Scientific (M-1157) and Sigma-Aldrich (C4859), respectively.

### Dual luciferase assay

HeLa, 293T, and C33a cells were seeded in 24-well culture plates a day before transfection and transfected with TEAD-binding element reporter (8xGTIIC-luc), the control Renilla luciferase plasmid (pRL-SV40), and the plasmids expressing PTPN14 for 24 hours (HeLa and 293T cells) or 48 hours (C33a cells). After removing the growth medium, plates were washed with PBS to remove any detached cells and residual growth medium. The cells were lysed with 1X passive lysis buffer by rocking at room temperature, and the lysates were transferred to 96-well luminescence plates. Luciferase activity was measured using the Dual-Luciferase Reporter Assay System (Promega) according to the manufacturer’s instructions.

### Immunohistochemistry

Immunohistochemistry was performed on HeLa cells using an antibody against YAP/TAZ (8418S; Cell Signaling), PTPN14 (sc-373766; Santa Cruz Biotechnology), Alexa Fluor 594 (A11012; Thermo Fisher), and Alexa Fluor 488 (A11001; Thermo Fisher), which were used as fluorescent secondary antibodies compatible with the primary antibody. Cells were cultured on the confocal dishes and transfected with appropriate genes using Lipofectamine 3000 for 24 hours. The cells were then washed with PBS containing 0.1% bovine serum albumin and incubated with 4% paraformaldehyde for 20 minutes to fix them onto the dishes. To allow antibodies to pass through the cell membrane and to minimize nonspecific interactions, the cells were incubated with PBS-supplemented 10% goat serum and 0.3% Triton X-100 for 30 minutes. Then, the cells were incubated overnight at 4 °C with the primary antibodies against YAP and PTPN14 in 10% goat serum. The plates were washed three times for 5 minutes with PBS containing 0.1% bovine serum albumin prior to incubation with fluorescent secondary antibodies. The next day, the cells were washed and incubated with fluorescent dye–conjugated secondary antibodies for 1 hour at room temperature. ProLong Gold antifade reagent with DAPI (P36931; Thermo Fisher) was used to mount immunostained cells and to stain cell nuclei. Then, the plates were covered with 0.8-mm coverslips and viewed using a fluorescence microscope IX2-UCB (Olympus). The numbers of total and YAP/TAZ-nuclear excluded cells were counted manually based on immunofluorescence images for calculating the percentage of cellular localization of HeLa cells in cytoplasm and nucleus. Data are expressed as the means ± SD of four immunofluorescence images from two independent experiments.

### Cell proliferation assays

For cell growth and survival assays, stable HeLa and HaCaT cells were seeded in a 48-well plate at 2,000 cells per well and were cultured for 0–4 days in 200 μL DMEM media containing 10% fetal bovine serum and 1% antibiotic-antimycotic solution without medium change. C33a cells were transfected with appropriate genes for 24 hours using Lipofectamine 3000 prior to seeding cells in a 48-well plate at 2,000 cells per well for the short-term proliferation assay. Cell proliferation was analyzed using CellTiter-Glo luminescent cell viability assay kit (Promega). After 10 minutes of incubation, luminescence was measured with a luminometer Wallac Victor X3 (Perkin Elmer).

### Clonogenic assay

Stable HaCaT cells were seeded in a 6-well plate at 4 × 10^4^ cells per well in triplicate and then grown for 6 days. Stable HeLa cells were seeded in a 6-well plate at 1,000 cells per well in triplicate and then cultured for 15 days. Medium was changed every 5 days. For crystal violet staining, PBS-washed plates were incubated in 4% paraformaldehyde in PBS for 20 minutes and then stained with 0.5% crystal violet solution in filtered distilled water for 20 minutes. Plates were washed subsequently with filtered distilled water and dried, and images were captured using a camera. Stained area of cells or colonies was quantified using ImageJ Plugin. The number of stained cells or colonies was photographed and counted.

### Migration and invasion assay

The migration and invasion assay was performed using Costar Transwell chambers containing an 8.0-μm polycarbonate membrane (3422; Costar). HeLa cells stably expressing wild-type and the SQA mutant PTPN14, HaCaT cells stably expressing wild-type and the AA mutant HPV18 E7, and C33a cells transiently expressing wild-type and the SQA mutant PTPN14 were collected and suspended at a density of 3 × 10^4^ cells/mL, 3 × 10^5^ cells/mL, and 1 × 10^5^ cells/mL for migration, respectively, in 100 μL of serum-free DMEM medium. Stable HeLa cells were also collected and suspended at a density of 1.5 × 10^6^ cells/mL for the invasion assay in 100 μL of the same medium. The media containing 10% FBS was subsequently added to the wells of the plates. For the invasion assay, a thin layer of Matrigel (354234; Corning) was added to the membrane matrix prior to seeding. On top of the inserts and Matrigel-coated inserts, 100 μL of prepared cells was added. The chamber plate was incubated for 24 hours in a cell incubator at a temperature of 37 °C and in an atmosphere of 5% CO_2_. The cells that invaded into the membrane were fixed with 4% paraformaldehyde (Biosesang) in PBS for 30 minutes and stained with 0.5% crystal violet (Tokyo Chemical Industry) for 20 minutes. Migrated and invaded cells were observed with a microscope Eclipse Ts2 (Nikon), and images were obtained to count the total number of migrated and invaded cells.

### Quantitative real-time polymerase chain reaction (qRT-PCR)

RNA isolation was performed with the easy-spin total RNA extraction kit (iNtRON) according to manufactures’ instructions. Extracted RNA was utilized for cDNA synthesis using MMLV reverse transcriptase (Promega) as described in the manufactures’ protocol. cDNAs were amplified by CFX Connect RT-PCR detection system (Bio-Rad) using specific primers and the EvaGreen RT-PCR kit (SolGent) to determine mRNA levels. Experiments were performed in triplicate, and each value was normalized to β-Actin. Primer sequences are listed in S4 Table.

## Supporting information

S1 Fig Confirmation of complex formation by SEC. Copurified protein samples were subjected to a HiLoad 26/600 Superdex 75 pg gel filtration column for final purification, and the resulting fractions were loaded onto SDS-PAGE and visualized by Coomassie blue staining together with size marker. (A) PTPN14 and HPV18 E7; (B) PTPN21 and HPV18 E7; (C) PTPN14 and E7 proteins from five different HPV genotypes. HPV, human papilloma virus; PTPN14, nonreceptor-type protein tyrosine phosphatase 14; SDS-PAGE, sodium dodecyl sulfate–polyacrylamide gel electrophoresis; SEC, size-exclusion chromatography.
(TIF)

S2 Fig Structural analysis of the PTPN14 PTP domain between apo and HPV18 E7–bound forms. (A) C_α_ traces of two forms of the PTPN14 PTP domain are superimposed and shown in a stereo view. (B) Conformation of the catalytic pocket residues are structurally aligned and shown in a stereo view. Residues constituting the phosphate-binding loop and the WPD loop are present in sticks and labeled. HPV, human papilloma virus; PTP, protein tyrosine phosphatase; PTPN14, nonreceptor-type PTP 14; WPD, tryptophan-proline-aspartate.
(TIF)

S3 Fig Intermolecular hydrogen bonds between PTPN14 and HPV18 E7. Direct or water (represented as red spheres)-mediated hydrogen bonds between PTPN14 (violet) and HPV E7 (green) shown in sticks are indicated by dashed lines. Labeled are residues involved in hydrogen bonds. HPV, human papilloma virus; PTPN14, nonreceptor-type protein tyrosine phosphatase 14.
(TIF)

S4 Fig Homodimerization of the C-terminal domain of HPV18 E7. (A) Intermolecular interaction between Flag-tagged and HA-tagged C-terminal domain of HPV18 E7. The 293T cells were transfected with the indicated plasmids for 24 hours, and the interaction was examined by coimmunoprecipitation and immunoblotting. (B) Stereo view of packing of PTPN14 and HPV18 E7 molecules in crystals. Dimerization between symmetry-related HPV18 E7 molecules is indicated by a dashed rectangle. (C) Structural comparison between the E7 C-terminal domain from three different genotypes of HPV. C_α_ traces represent proteins, and red circles indicate coordinated zinc ions. HA, hemagglutinin; HPV, human papilloma virus; PTPN14, nonreceptor-type protein tyrosine phosphatase 14.
(TIF)

S5 Fig Sequence alignment. The sequence of the HPV18 E7–binding interface of PTPN14 is aligned with those of the corresponding regions of the PTP D1 domain (A) or PTP D2 domain (B) of receptor-type PTP proteins, as in Fig 4A. HPV, human papilloma virus; PTP, protein tyrosine phosphatase; PTPN14, nonreceptor-type PTP 14.
(TIF)

S6 Fig Structural alignment. The PTP domain of PTPN14 bound to HPV18 E7 is superimposed onto those of four nonreceptor-type PTP proteins (PTPN7, PTPN9, SHP2, and PTPN12) and two receptor-type PTP proteins (PTPσ and PTPε), as in Fig 4B. PTP, protein tyrosine phosphatase.
(TIF)

S7 Fig Binding profiles of the E7 C-terminal domain from variable HPV genotypes. (A) Sequences of the C-terminal domain of E7 from six HPV genotypes are aligned. The secondary structures of HPV18 E7 are shown at the top. Conserved residues are shaded in orange. Six residues in HPV18 E7 that play a key role in the intermolecular interaction with PTPN14 are marked by red arrows. (B) SEC-MALS analysis confirms the complex formation between the PTPN14 PTP domain and the E7 C-terminal domain from the indicated HPV genotypes. Solid lines, molar masses in kg/mol; dotted lines, refractive indexes. Constructs are listed in S1 Table. The numerical data are included in S1 Data. HPV, human papilloma virus; PTP, protein tyrosine phosphatase; PTPN14, nonreceptor-type PTP 14; SEC-MALS, size-exclusion chromatography–multiangle light scattering.
(TIF)

S8 Fig Trials for purification of the HPV E7 C-terminal domain alone. His_6_–MBP-tagged E7 C-terminal domain protein samples from six different HPV genotypes were treated with TEV protease for 16 hours at 4 °C and then analyzed using a HiLoad 26/600 Superdex 75 pg gel filtration column. The peak fractions in each analysis were analyzed and visualized by SDS-PAGE and Coomassie staining. Red arrows indicate the fractions containing HPV18 E7(54–105) separated from His_6_–MBP. HPV, human papillomavirus; I, input; MBP, maltose binding protein; S, size marker; SDS-PAGE, sodium dodecyl sulfate–polyacrylamide gel electrophoresis; TEV, tobacco etch virus.
(TIF)

S9 Fig Thermal shift assay. Two PTPN14 samples were diluted to 0.6 mg/mL and prepared in a 20 μL reaction buffer containing 50 mM Tris-HCl (pH 7.5), 200 mM NaCl, 2 mM dithiothreitol, and 8X diluted SYPRO orange fluorescent dye (Applied Biosystems). Samples were pipetted into 8-well PCR tubes and sealed with optical flat strips (Bio-Rad). All experiments were performed using a Bio-Rad CFX96 real-time system, and the ROX reporter was chosen to collect fluorescent emission signals. Temperature was held for 5 sec at 0.5-degree intervals from 25 °C to 99 °C. The numerical data are included in S1 Data. PCR, polymerase chain reaction; PTPN14, nonreceptor-type protein tyrosine phosphatase 14; ROX, carboxy-X-rhodamine.
(TIF)

S10 Fig Cellular assay using 293T cells. (A) Protein levels of transiently expressed wild-type and the SQA mutant PTPN14 were not affected by 20 μM MG132 treatment for 8 hours in 293T cells. (B) Interactions of wild-type and the SQA mutant PTPN14 and Hippo components YAP and LATS1 transiently expressed in 293T cells were examined by coimmunoprecipitation and immunoblotting. (C) Transcriptional activity of YAP in 293T cells with or without wild-type or the SQA mutant PTPN14 was measured by quantifying luciferase activity. The YAP activity was suppressed significantly by transient expression of both the PTPN14 constructs. ***P < 0.001 in the Student *t* test. The numerical data are included in S1 Data. LATS1, large tumor suppressor 1; ns, not significant; PTPN14, nonreceptor-type protein tyrosine phosphatase 14; SQA, F1044S, G1055Q, and E1095A; YAP, yes-associated protein.
(TIF)

S11 Fig Effects of E7 binding-defective mutations of PTPN14 were analyzed in C33a cells. The indicated PTPN14 constructs were transiently expressed in C33a cells. *P < 0.05; **P < 0.01; ***P < 0.001 in the Student *t* test. The numerical data are included in S1 Data. (A) Transcriptional activity of YAP in C33a cells with or without wild-type or the SQA mutant PTPN14 was measured by quantifying luciferase activity. The YAP activity was suppressed significantly by transient expression of both the PTPN14 constructs. (B) Growth curves of C33a cells transiently expressing empty-vector control (Mock) or wild-type or the SQA mutant PTPN14 are compared from day 0 to day 3. (C) Motility of C33a cells with or without wild-type or the SQA mutant PTPN14 was analyzed and compared. (Left) Representative cell images stained with crystal violet. The scale bars indicate 20 μm. (Right) The number of migrated cells were quantified as bar graphs. ns, not significant; PTPN14, nonreceptor-type protein tyrosine phosphatase 14; SQA, F1044S, G1055Q, and E1095A; YAP, yes-associated protein.
(TIF)

S1 Table Constructs of recombinant proteins used in SEC-MALS experiments. Residues that are derived from tags or translation-initiating methionine are marked in red. SEC-MALS, size-exclusion chromatography–multiangle light scattering.
(DOCX)

S2 Table Constructs of recombinant proteins prepared for crystallization. Residues that are derived from tags or translation-initiating methionine are marked in red. Residues removed during purification are underlined.
(DOCX)

S3 Table Constructs used in cellular assays for transient or stable expression. (XLSX)

S4 Table Primers used in qRT-PCR. qRT-PCR, quantitative real-time polymerase chain reaction.
(XLSX)

S1 Data Numerical data underlying Figs 1A, 4D, 6B–6E and 7A–7F and S7B, S9, S10C and S11A–S11C Figs. (XLSX)

## Abbreviations

AA: R84A and L91A
CHX: cycloheximide
CR: conserved region
CTGF: connective tissue growth factor
CYR61: cysteine-rich angiogenic inducer 61
HA: hemagglutinin
HDAC: histone deacetylase
HPV: human papillomavirus
ITC: isothermal titration calorimetry
KRT: Keratin
LATS1: large tumor suppressor 1
MALS: multiangle light scattering
MBP: maltose binding protein
Mn: number-average molar mass
n.s.: not significant
NTA: nitrilotriacetic acid
PAGE: polyacrylamide gel electrophoresis
pRb: retinoblastoma protein
PTP: protein tyrosine phosphatase
PTPN14: nonreceptor-type protein tyrosine phosphatase 14
qRT-PCR: quantitative real-time polymerase chain reaction
RMSD: root-mean-square deviation
SDS: sodium dodecyl sulfate
SEC: size-exclusion chromatography
SQA: F1044S, G1055Q, and E1095A
TAZ: tafazzin
TEV: tobacco etch virus
WCL: whole-cell lysate
WPD: tryptophan-proline-aspartate
YAP: yes-associated protein
