# Supplementary figures and images for: Structural basis for recognition of the tumor suppressor protein PTPN14 by the oncoprotein E7 of human papillomavirus
Source: PLoS Biol. 2019 Jul 19;17(7):e3000367. doi: 10.1371/journal.pbio.3000367 (PMC6668832; doi:10.1371/journal.pbio.3000367)

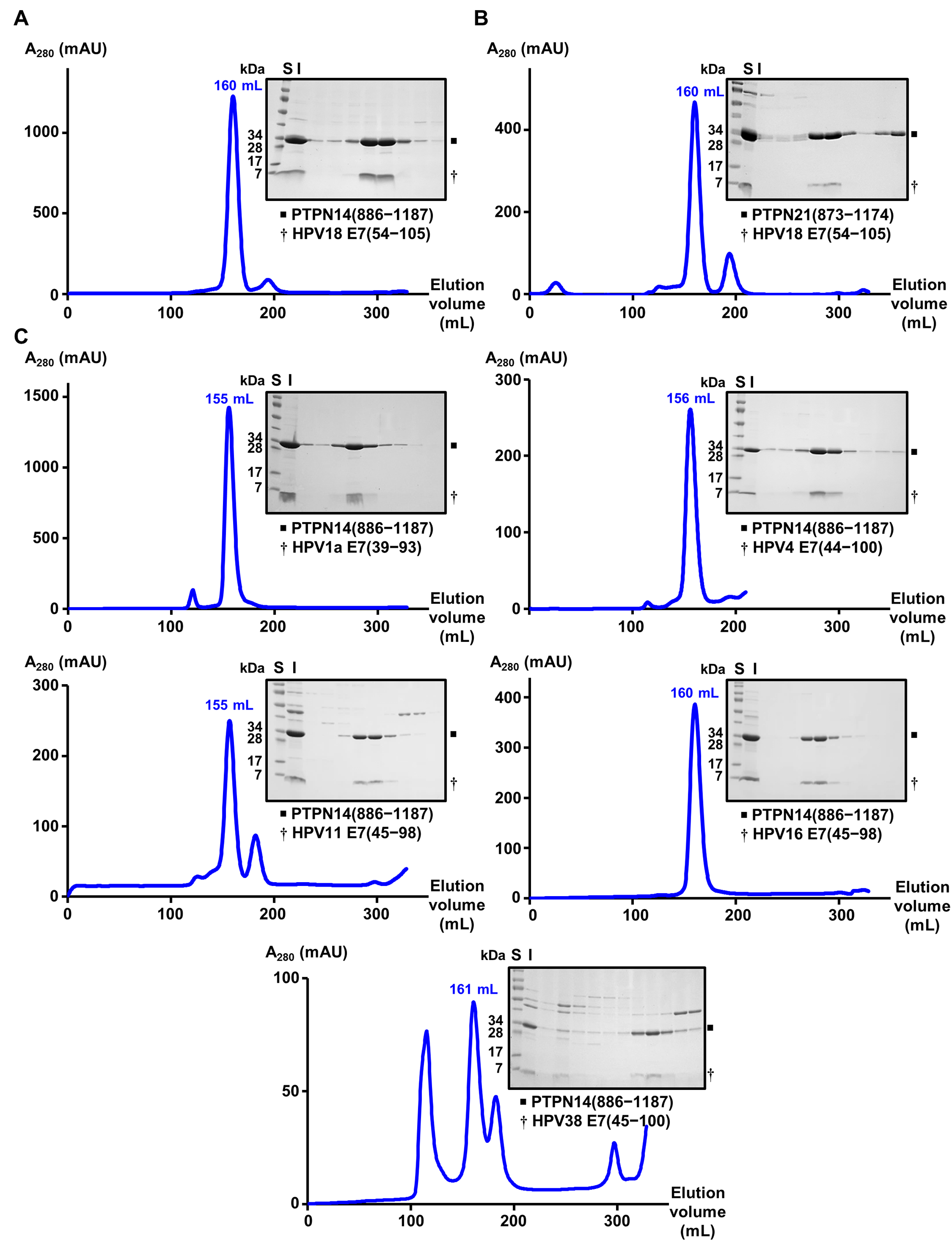

Supplement: S1 Fig — Copurified protein samples were subjected to a HiLoad 26/600 Superdex 75 pg gel filtration column for final purification, and the resulting fractions were loaded onto SDS-PAGE and visualized by Coomassie blue staining together with size marker. (A) PTPN14 and HPV18 E7; (B) PTPN21 and HPV18 E7; (C) PTPN14 and E7 proteins from five different HPV genotypes. HPV, human papilloma virus; PTPN14, nonreceptor-type protein tyrosine phosphatase 14; SDS-PAGE, sodium dodecyl sulfate–polyacrylamide gel electrophoresis; SEC, size-exclusion chromatography. (TIF) [file pbio.3000367.s001.tif]

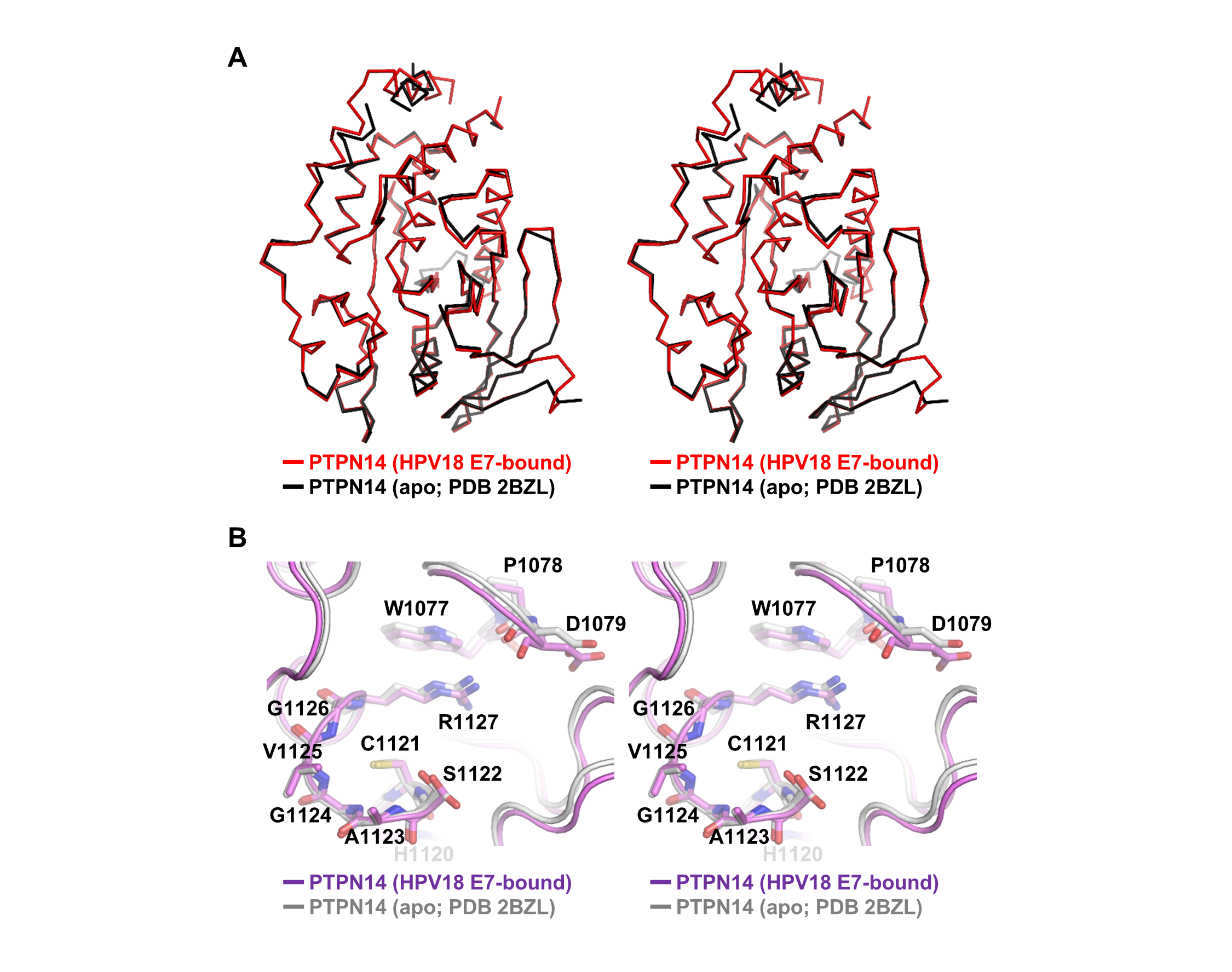

Supplement: S2 Fig — (A) Cα traces of two forms of the PTPN14 PTP domain are superimposed and shown in a stereo view. (B) Conformation of the catalytic pocket residues are structurally aligned and shown in a stereo view. Residues constituting the phosphate-binding loop and the WPD loop are present in sticks and labeled. HPV, human papilloma virus; PTP, protein tyrosine phosphatase; PTPN14, nonreceptor-type PTP 14; WPD, tryptophan-proline-aspartate. (TIF) [file pbio.3000367.s002.tif]

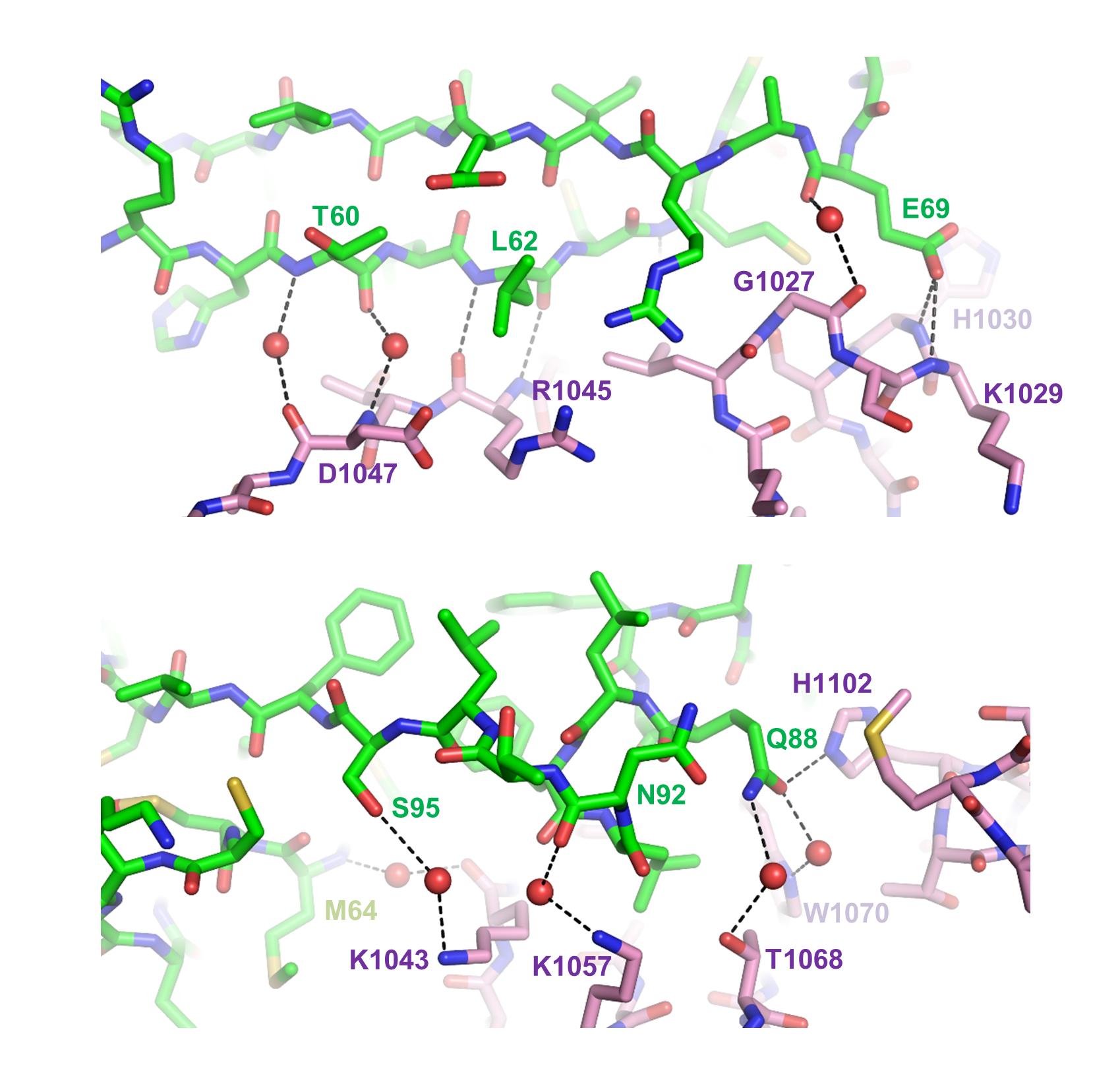

Supplement: S3 Fig — Direct or water (represented as red spheres)-mediated hydrogen bonds between PTPN14 (violet) and HPV E7 (green) shown in sticks are indicated by dashed lines. Labeled are residues involved in hydrogen bonds. HPV, human papilloma virus; PTPN14, nonreceptor-type protein tyrosine phosphatase 14. (TIF) [file pbio.3000367.s003.tif]

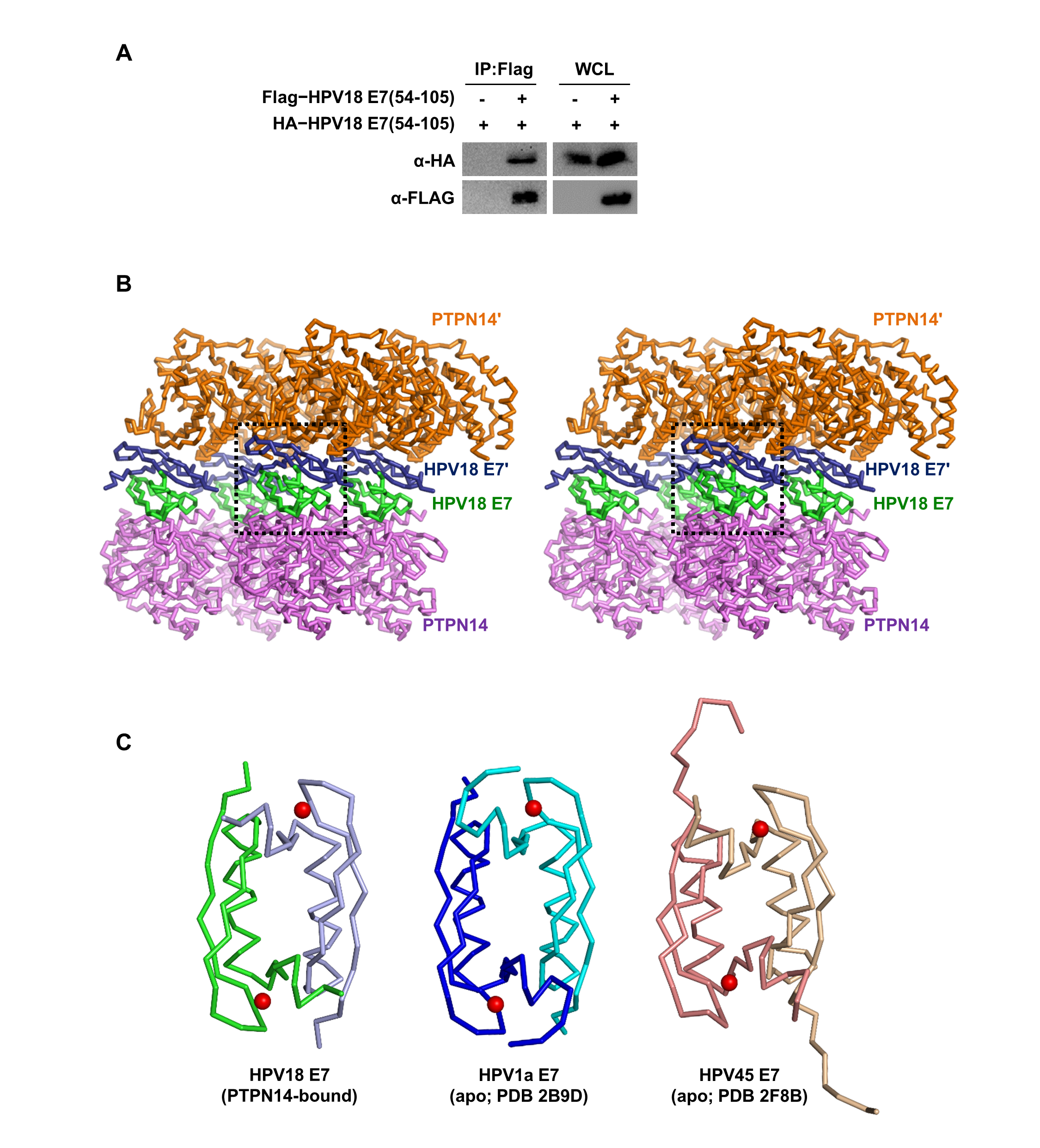

Supplement: S4 Fig — (A) Intermolecular interaction between Flag-tagged and HA-tagged C-terminal domain of HPV18 E7. The 293T cells were transfected with the indicated plasmids for 24 hours, and the interaction was examined by coimmunoprecipitation and immunoblotting. (B) Stereo view of packing of PTPN14 and HPV18 E7 molecules in crystals. Dimerization between symmetry-related HPV18 E7 molecules is indicated by a dashed rectangle. (C) Structural comparison between the E7 C-terminal domain from three different genotypes of HPV. Cα traces represent proteins, and red circles indicate coordinated zinc ions. HA, hemagglutinin; HPV, human papilloma virus; PTPN14, nonreceptor-type protein tyrosine phosphatase 14. (TIF) [file pbio.3000367.s004.tif]

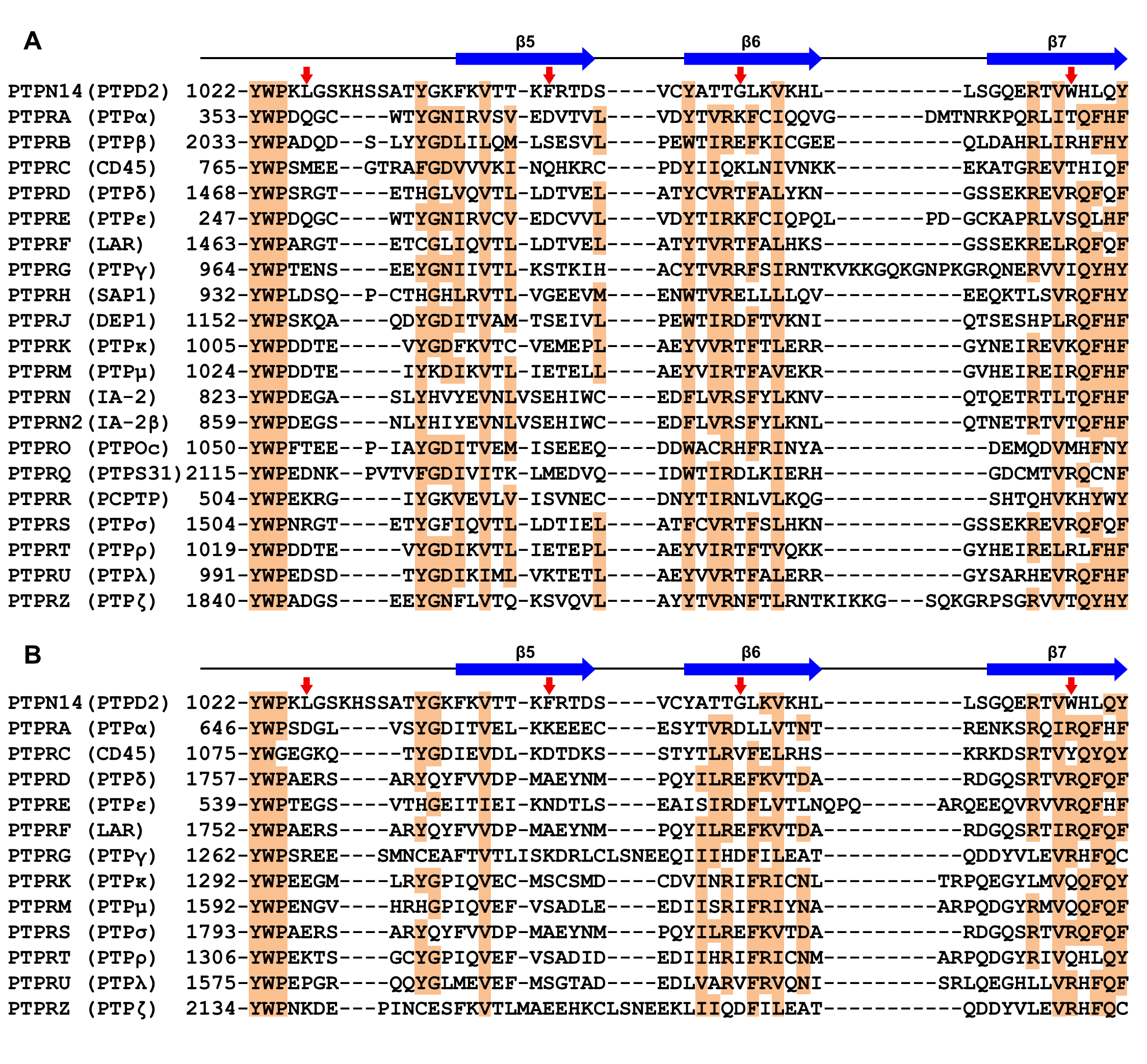

Supplement: S5 Fig — The sequence of the HPV18 E7–binding interface of PTPN14 is aligned with those of the corresponding regions of the PTP D1 domain (A) or PTP D2 domain (B) of receptor-type PTP proteins, as in Fig 4A. HPV, human papilloma virus; PTP, protein tyrosine phosphatase; PTPN14, nonreceptor-type PTP 14. (TIF) [file pbio.3000367.s005.tif]

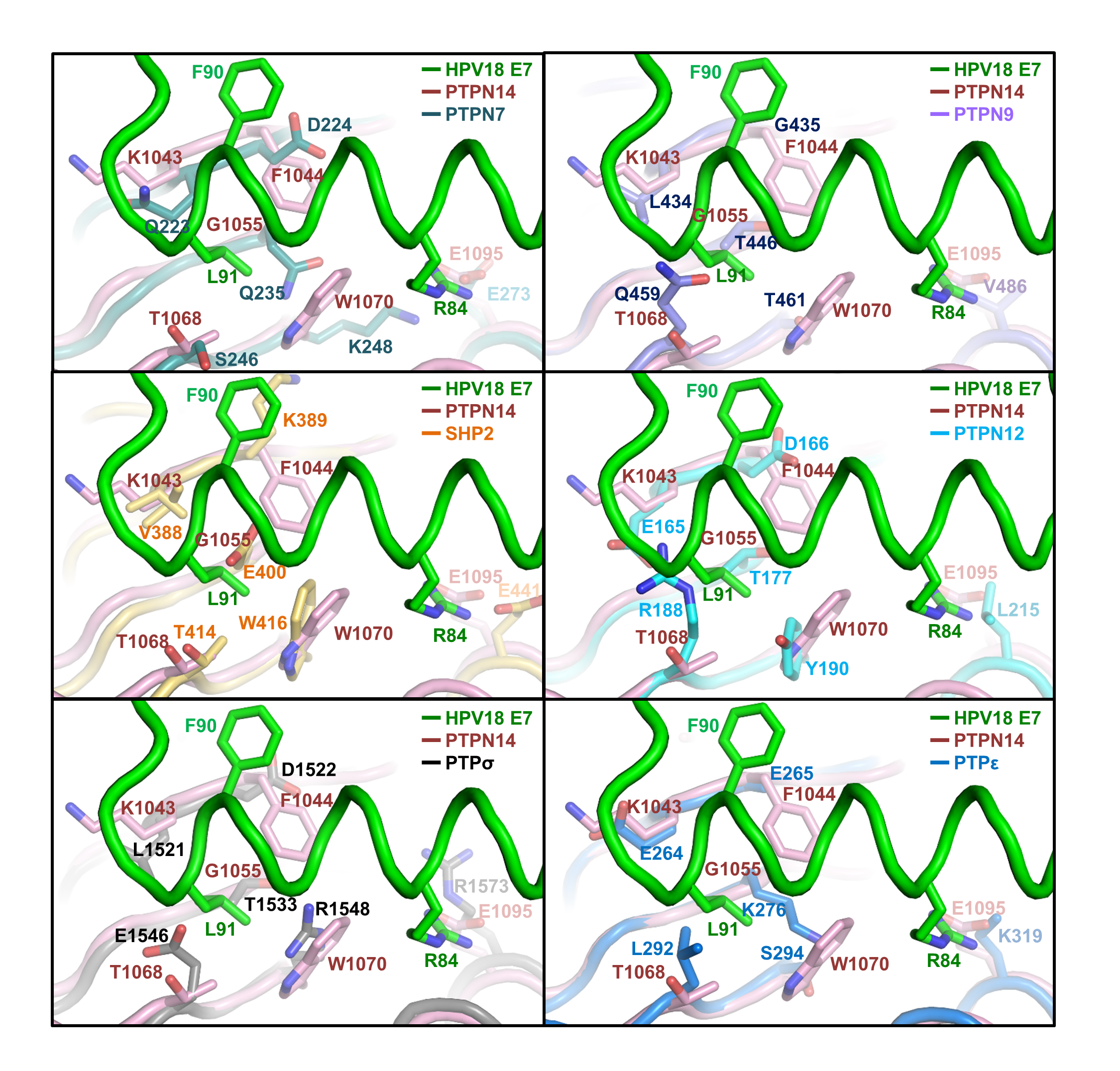

Supplement: S6 Fig — The PTP domain of PTPN14 bound to HPV18 E7 is superimposed onto those of four nonreceptor-type PTP proteins (PTPN7, PTPN9, SHP2, and PTPN12) and two receptor-type PTP proteins (PTPσ and PTPε), as in Fig 4B. PTP, protein tyrosine phosphatase. (TIF) [file pbio.3000367.s006.tif]

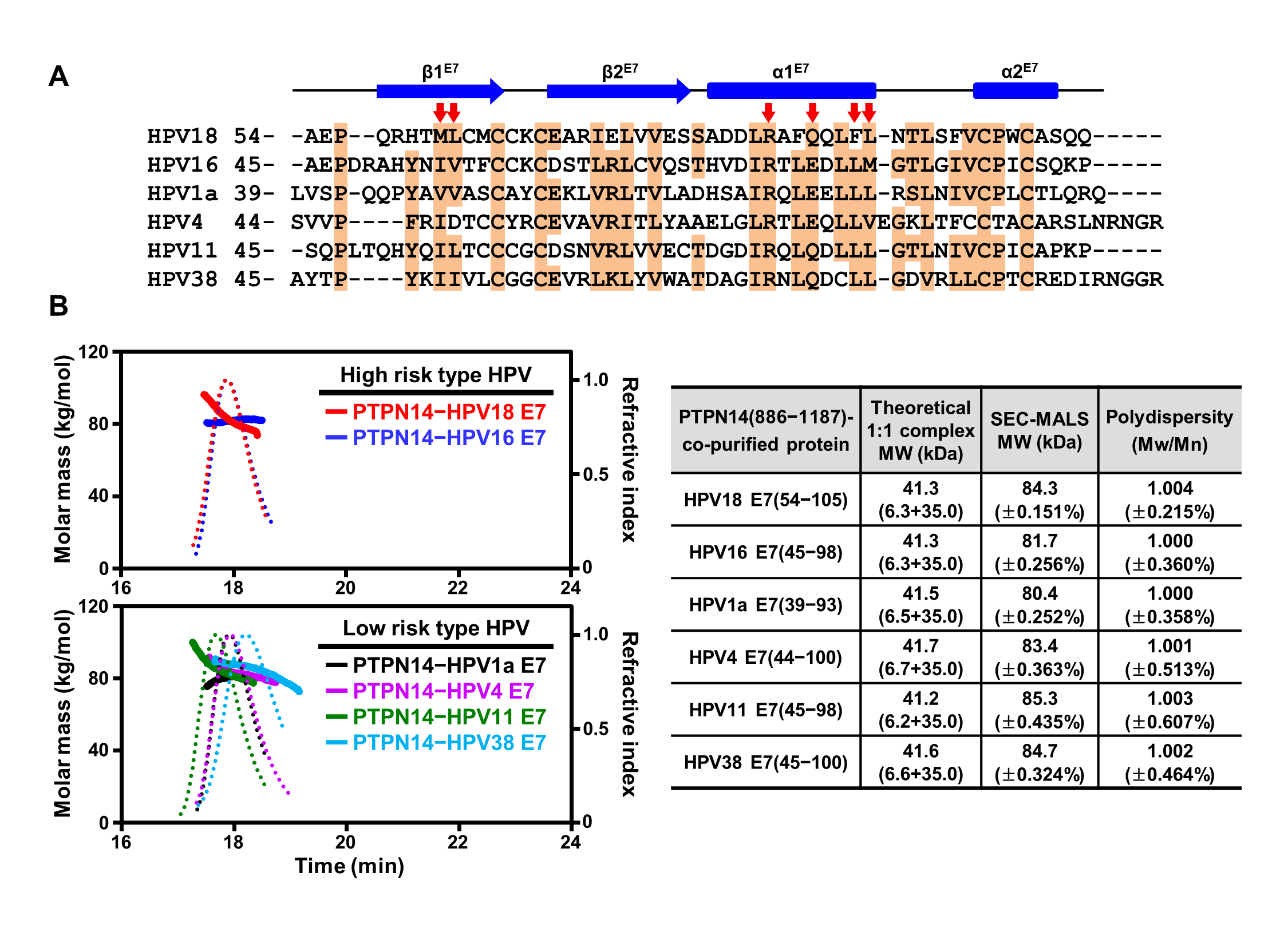

Supplement: S7 Fig — (A) Sequences of the C-terminal domain of E7 from six HPV genotypes are aligned. The secondary structures of HPV18 E7 are shown at the top. Conserved residues are shaded in orange. Six residues in HPV18 E7 that play a key role in the intermolecular interaction with PTPN14 are marked by red arrows. (B) SEC-MALS analysis confirms the complex formation between the PTPN14 PTP domain and the E7 C-terminal domain from the indicated HPV genotypes. Solid lines, molar masses in kg/mol; dotted lines, refractive indexes. Constructs are listed in S1 Table. The numerical data are included in S1 Data. HPV, human papilloma virus; PTP, protein tyrosine phosphatase; PTPN14, nonreceptor-type PTP 14; SEC-MALS, size-exclusion chromatography–multiangle light scattering. (TIF) [file pbio.3000367.s007.tif]

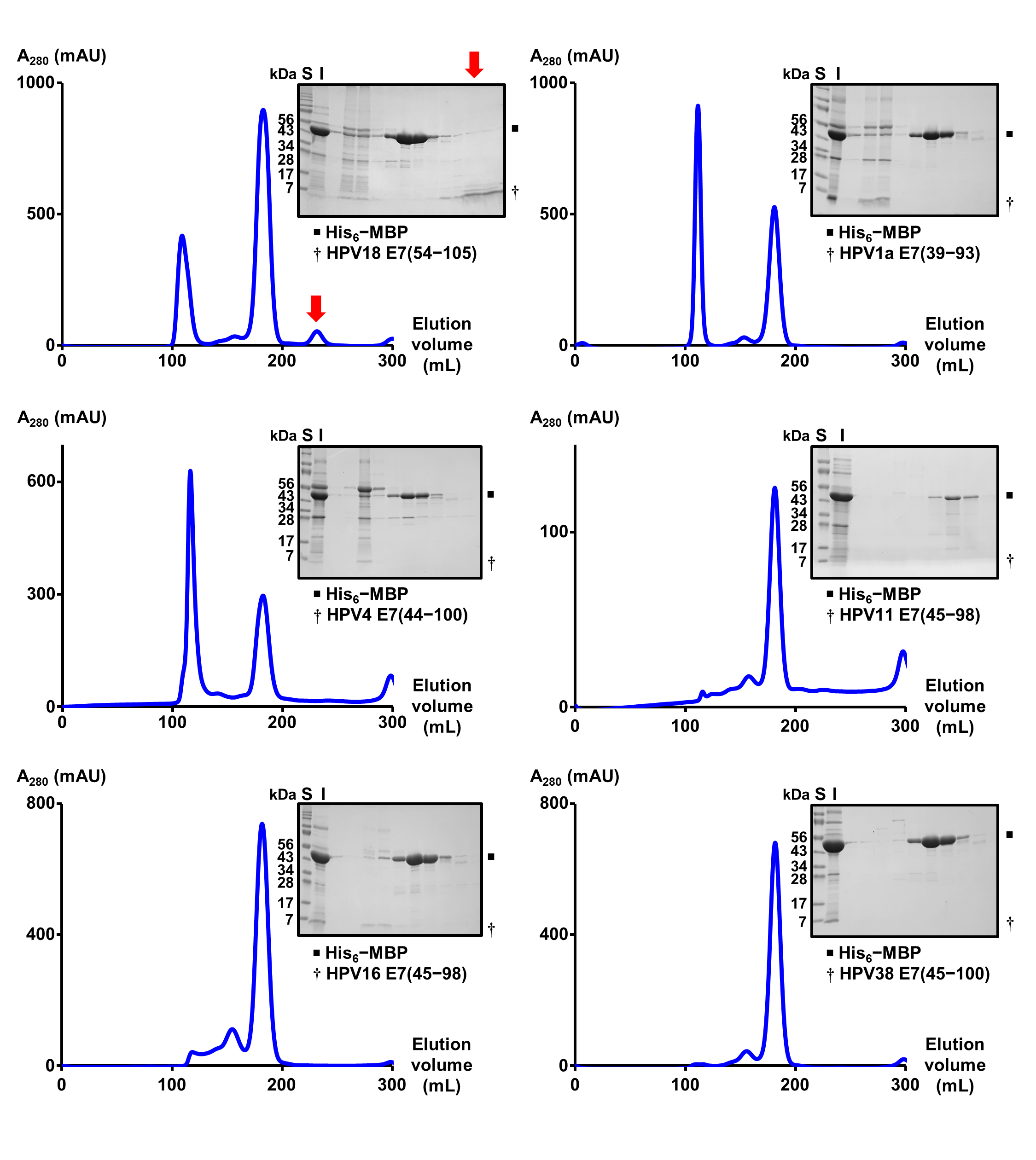

Supplement: S8 Fig — His6–MBP-tagged E7 C-terminal domain protein samples from six different HPV genotypes were treated with TEV protease for 16 hours at 4 °C and then analyzed using a HiLoad 26/600 Superdex 75 pg gel filtration column. The peak fractions in each analysis were analyzed and visualized by SDS-PAGE and Coomassie staining. Red arrows indicate the fractions containing HPV18 E7(54–105) separated from His6–MBP. HPV, human papillomavirus; I, input; MBP, maltose binding protein; S, size marker; SDS-PAGE, sodium dodecyl sulfate–polyacrylamide gel electrophoresis; TEV, tobacco etch virus. (TIF) [file pbio.3000367.s008.tif]

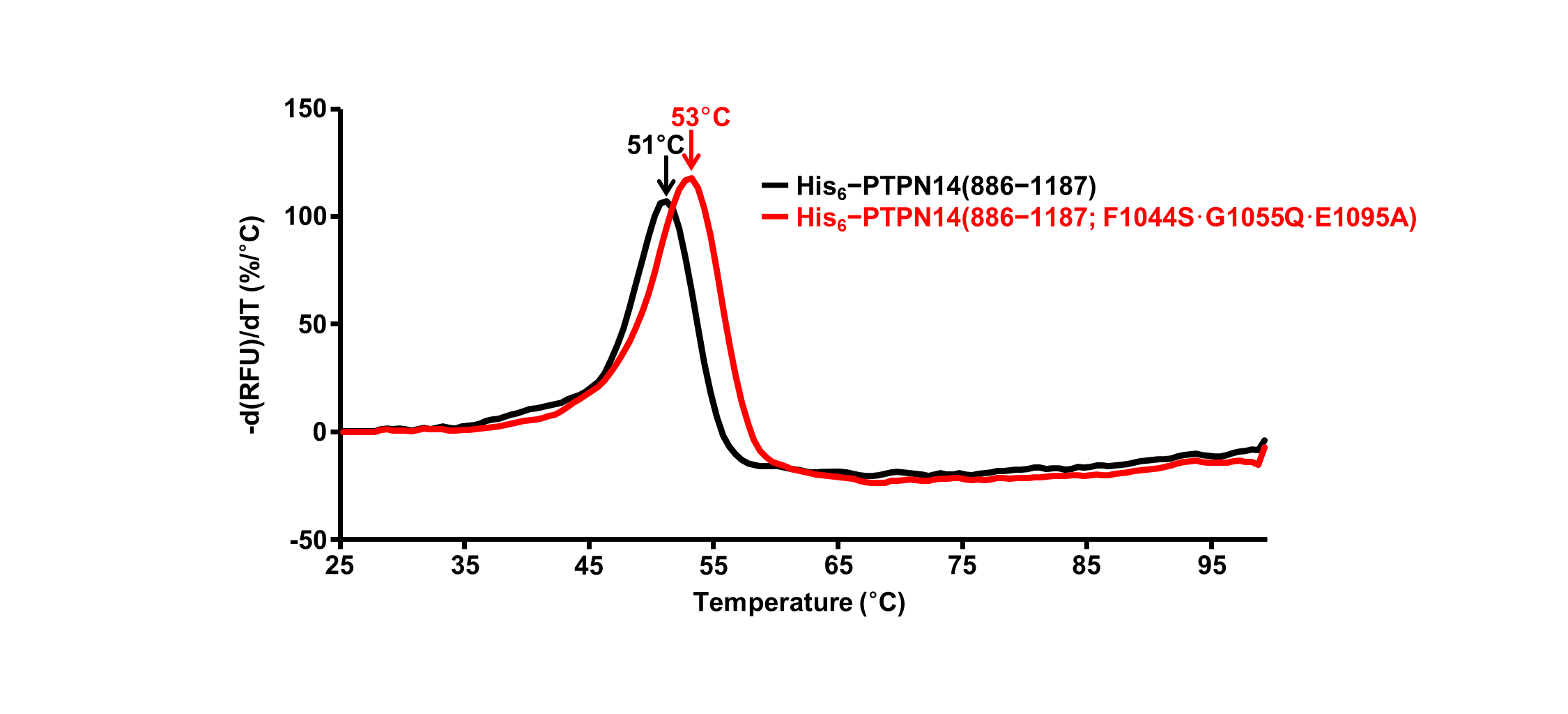

Supplement: S9 Fig — Two PTPN14 samples were diluted to 0.6 mg/mL and prepared in a 20 μL reaction buffer containing 50 mM Tris-HCl (pH 7.5), 200 mM NaCl, 2 mM dithiothreitol, and 8X diluted SYPRO orange fluorescent dye (Applied Biosystems). Samples were pipetted into 8-well PCR tubes and sealed with optical flat strips (Bio-Rad). All experiments were performed using a Bio-Rad CFX96 real-time system, and the ROX reporter was chosen to collect fluorescent emission signals. Temperature was held for 5 sec at 0.5-degree intervals from 25 °C to 99 °C. The numerical data are included in S1 Data. PCR, polymerase chain reaction; PTPN14, nonreceptor-type protein tyrosine phosphatase 14; ROX, carboxy-X-rhodamine. (TIF) [file pbio.3000367.s009.tif]

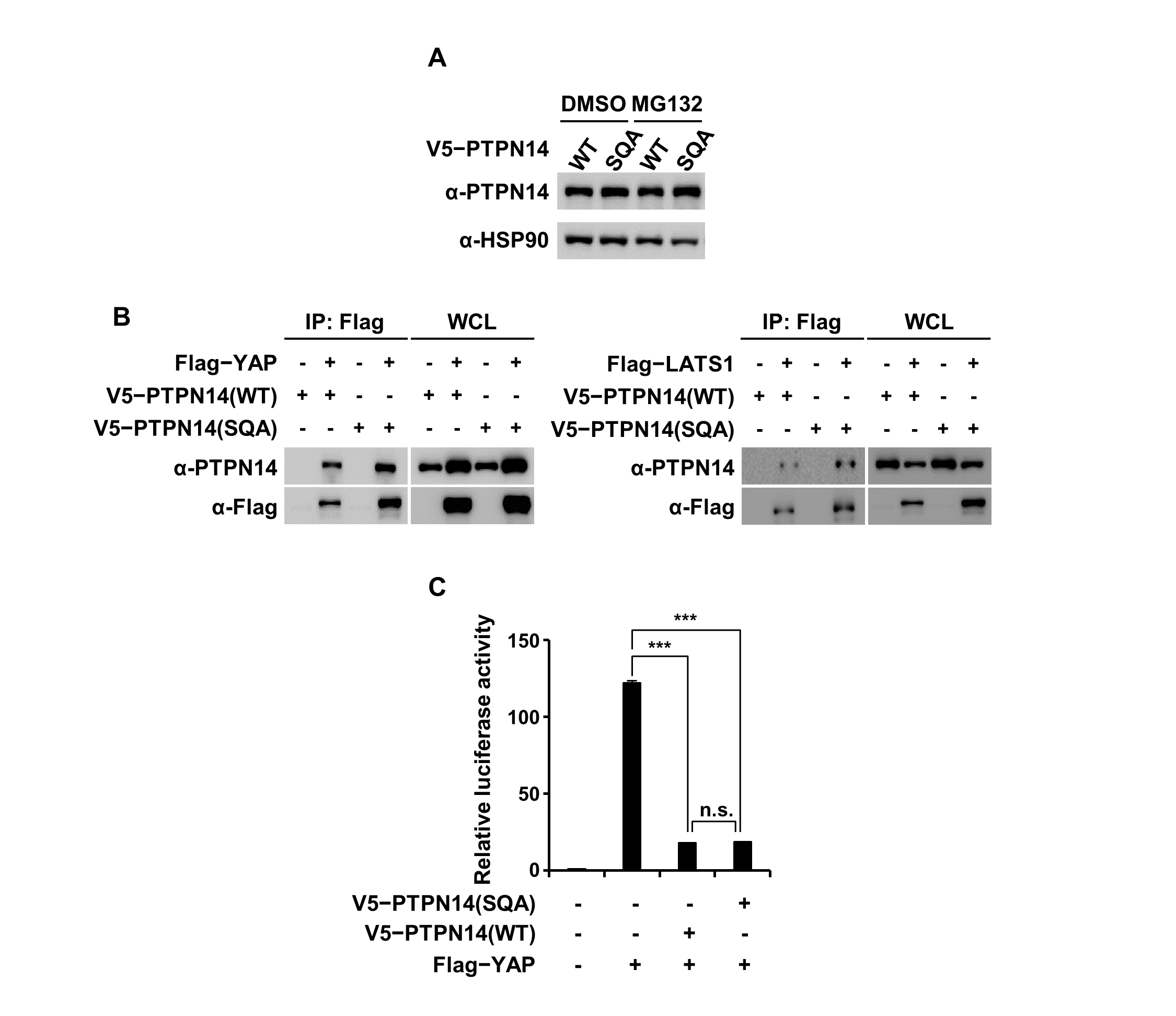

Supplement: S10 Fig — (A) Protein levels of transiently expressed wild-type and the SQA mutant PTPN14 were not affected by 20 μM MG132 treatment for 8 hours in 293T cells. (B) Interactions of wild-type and the SQA mutant PTPN14 and Hippo components YAP and LATS1 transiently expressed in 293T cells were examined by coimmunoprecipitation and immunoblotting. (C) Transcriptional activity of YAP in 293T cells with or without wild-type or the SQA mutant PTPN14 was measured by quantifying luciferase activity. The YAP activity was suppressed significantly by transient expression of both the PTPN14 constructs. ***P < 0.001 in the Student t test. The numerical data are included in S1 Data. LATS1, large tumor suppressor 1; ns, not significant; PTPN14, nonreceptor-type protein tyrosine phosphatase 14; SQA, F1044S, G1055Q, and E1095A; YAP, yes-associated protein. (TIF) [file pbio.3000367.s010.tif]

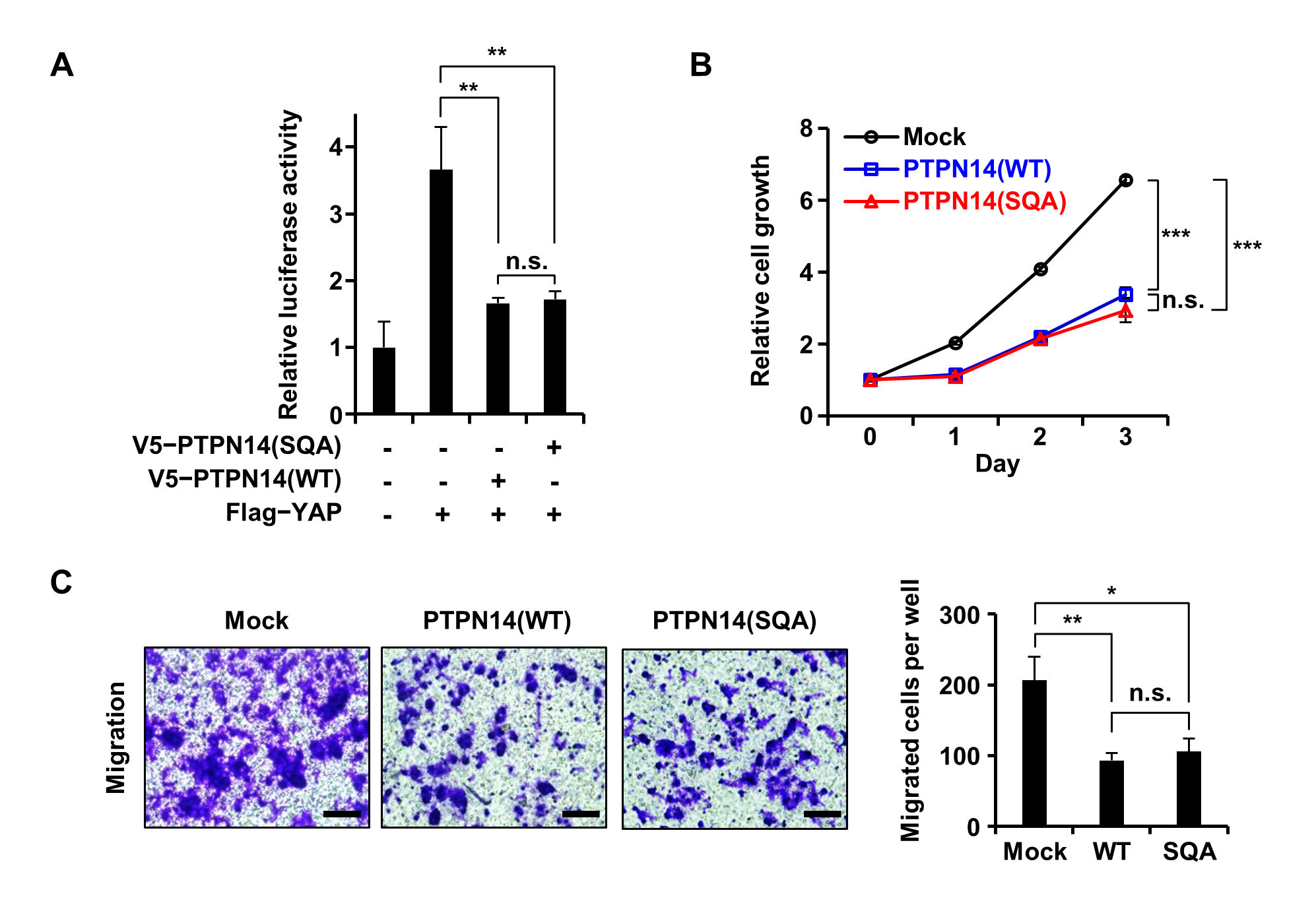

Supplement: S11 Fig — The indicated PTPN14 constructs were transiently expressed in C33a cells. *P < 0.05; **P < 0.01; ***P < 0.001 in the Student t test. The numerical data are included in S1 Data. (A) Transcriptional activity of YAP in C33a cells with or without wild-type or the SQA mutant PTPN14 was measured by quantifying luciferase activity. The YAP activity was suppressed significantly by transient expression of both the PTPN14 constructs. (B) Growth curves of C33a cells transiently expressing empty-vector control (Mock) or wild-type or the SQA mutant PTPN14 are compared from day 0 to day 3. (C) Motility of C33a cells with or without wild-type or the SQA mutant PTPN14 was analyzed and compared. (Left) Representative cell images stained with crystal violet. The scale bars indicate 20 μm. (Right) The number of migrated cells were quantified as bar graphs. ns, not significant; PTPN14, nonreceptor-type protein tyrosine phosphatase 14; SQA, F1044S, G1055Q, and E1095A; YAP, yes-associated protein. (TIF) [file pbio.3000367.s011.tif]
