# Supplementary material for: Structural basis for recognition of the tumor suppressor protein PTPN14 by the oncoprotein E7 of human papillomavirus
Source: PLoS Biol. 2019 Jul 19;17(7):e3000367. doi: 10.1371/journal.pbio.3000367 (PMC6668832; doi:10.1371/journal.pbio.3000367)
Supplement: S1 Table — Residues that are derived from tags or translation-initiating methionine are marked in red. SEC-MALS, size-exclusion chromatography–multiangle light scattering. (DOCX) [file pbio.3000367.s012.docx]

**Table S1. Constructs of recombinant proteins used in SEC-MALS experiments**

| **Protein** | **Residue (a.a)** | **Amino acid sequence** |
| --- | --- | --- |
| His_6_−PTPN14  (886−1187) | 323  (21+302) | MGSSHHHHHHSSGLVPRGSHMVDATRVPMDERFRTLKKKLEEGMVFTEYEQIPKKKANGIFSTAALPENAERSRIREVVPYEENRVELIPTKENNTGYINASHIKVVVGGAEWHYIATQGPLPHTCHDFWQMVWEQGVNVIAMVTAEEEGGRTKSHRYWPKLGSKHSSATYGKFKVTTKFRTDSVCYATTGLKVKHLLSGQERTVWHLQYTDWPDHGCPEDVQGFLSYLEEIQSVRRHTNSMLEGTKNRHPPIVVHCSAGVGRTGVLILSELMIYCLEHNEKVEVPMMLRLLREQRMFMIQTIAQYKFVYQVLIQFLQNSRLI |
| PTPN14  (886−1187) | 303  (1+302) | MVDATRVPMDERFRTLKKKLEEGMVFTEYEQIPKKKANGIFSTAALPENAERSRIREVVPYEENRVELIPTKENNTGYINASHIKVVVGGAEWHYIATQGPLPHTCHDFWQMVWEQGVNVIAMVTAEEEGGRTKSHRYWPKLGSKHSSATYGKFKVTTKFRTDSVCYATTGLKVKHLLSGQERTVWHLQYTDWPDHGCPEDVQGFLSYLEEIQSVRRHTNSMLEGTKNRHPPIVVHCSAGVGRTGVLILSELMIYCLEHNEKVEVPMMLRLLREQRMFMIQTIAQYKFVYQVLIQFLQNSRLI |
| PTPN21  (873−1174) | 305  (3+302) | SHMEVATRATNDERCKILEQRLEQGMVFTEYERILKKRLVDGECSTARLPENAERNRFQDVLPYDDVRVELVPTKENNTGYINASHIKVSVSGIEWDYIATQGPLQNTCQDFWQMVWEQGIAIIAMVTAEEEGGREKSFRYWPRLGSRHNTVTYGRFKITTRFRTDSGCYATTGLKMKHLLTGQERTVWHLQYTDWPEHGCPEDLKGFLSYLEEIQSVRRHTNSTSDPQSPNPPLLVHCSAGVGRTGVVILSEIMIACLEHNEVLDIPRVLDMLRQQRMMLVQTLCQYTFVYRVLIQFLKSSRLI |
| HPV18 E7  (54−105) | 55  (3+52) | GHMAEPQRHTMLCMCCKCEARIELVVESSADDLRAFQQLFLNTLSFVCPWCASQQ |
| HPV16 E7  (45−98) | 57  (3+54) | GHMAEPDRAHYNIVTFCCKCDSTLRLCVQSTHVDIRTLEDLLMGTLGIVCPICSQKP |
| HPV1a E7  (39−93) | 58  (3+55) | GHMLVSPQQPYAVVASCAYCEKLVRLTVLADHSAIRQLEELLLRSLNIVCPLCTLQRQ |
| HPV4 E7  (44−100) | 60  (3+57) | GHMSVVPFRIDTCCYRCEVAVRITLYAAELGLRTLEQLLVEGKLTFCCTACARSLNRNGR |
| HPV11 E7  (45−98) | 57  (3+54) | GHMSQPLTQHYQILTCCCGCDSNVRLVVECTDGDIRQLQDLLLGTLNIVCPICAPKP |
| HPV38 E7  (45−100) | 59  (3+56) | GHMAYTPYKIIVLCGGCEVRLKLYVWATDAGIRNLQDCLLGDVRLLCPTCREDIRNGGR |
