# Supplementary material for: Structural basis for recognition of the tumor suppressor protein PTPN14 by the oncoprotein E7 of human papillomavirus
Source: PLoS Biol. 2019 Jul 19;17(7):e3000367. doi: 10.1371/journal.pbio.3000367 (PMC6668832; doi:10.1371/journal.pbio.3000367)
Supplement: S2 Table — Residues that are derived from tags or translation-initiating methionine are marked in red. Residues removed during purification are underlined. (DOCX) [file pbio.3000367.s013.docx]

**Table S2. Constructs of recombinant proteins prepared for crystallization**

| **Protein** | **Amino acid sequence** |
| --- | --- |
| PTPN14(886−1187) | MVDATRVPMDERFRTLKKKLEEGMVFTEYEQIPKKKANGIFSTAALPENAERSRIREVVPYEENRVELIPTKENNTGYINASHIKVVVGGAEWHYIATQGPLPHTCHDFWQMVWEQGVNVIAMVTAEEEGGRTKSHRYWPKLGSKHSSATYGKFKVTTKFRTDSVCYATTGLKVKHLLSGQERTVWHLQYTDWPDHGCPEDVQGFLSYLEEIQSVRRHTNSMLEGTKNRHPPIVVHCSAGVGRTGVLILSELMIYCLEHNEKVEVPMMLRLLREQRMFMIQTIAQYKFVYQVLIQFLQNSRLI |
| His_6_−MBP−HPV18 E7 (54−105) | MGTSHHHHHHKIEEGKLVIWINGDKGYNGLAEVGKKFEKDTGIKVTVEHPDKLEEKFPQVAATGDGPDIIFWAHDRFGGYAQSGLLAEITPDKAFQDKLYPFTWDAVRYNGKLIAYPIAVEALSLIYNKDLLPNPPKTWEEIPALDKELKAKGKSALMFNLQEPYFTWPLIAADGGYAFKYENGKYDIKDVGVDNAGAKAGLTFLVDLIKNKHMNADTDYSIAEAAFNKGETAMTINGPWAWSNIDTSKVNYGVTVLPTFKGQPSKPFVGVLSAGINAASPNKELAKEFLENYLLTDEGLEAVNKDKPLGAVALKSYEEELAKDPRIAATMENAQKGEIMPNIPQMSAFWYAVRTAVINAASGRQTVDEALKDAQTNSSSNNNNNNNNNNLGLEDYDIPTTENLYFQGHMAEPQRHTMLCMCCKCEARIELVVESSADDLRAFQQLFLNTLSFVCPWCASQQ |
